# Morphology Design and Precision Control of Microneedles by PμSL 3D Printing

**DOI:** 10.3390/polym17101351

**Published:** 2025-05-15

**Authors:** Baoling Jia, Tiandong Xia, Yangtao Xu, Bei Li

**Affiliations:** 1 School of Materials Science and Engineering, Lanzhou University of Technology, Lanzhou 730050, China; 2 State Key Laboratory of Advanced Processing and Recycling of Non-Ferrous Metal Under the Province and the Ministry of Education, Lanzhou University of Technology, Lanzhou 730050, China

**Keywords:** microneedles, 3D printing, microneedle morphology, precision control

## Abstract

Microneedles (MNs) hold significant potential for applications in transdermal drug delivery and biosensing. However, when traditional 3D printing technology is used for their manufacture, a substantial deviation in output size occurs. The effects of various parameters on the morphology of MNs produced through microscale 3D printing remain unclear. This study investigated the relationship between the design and fabrication of acrylic resin MNs and their output forms via a projection microstereolithography (PµSL) technology system. Modifying the shape parameters and array configurations elucidates the causes of size deviation and proposes a control strategy. This is particularly significant for the prototyping and mold manufacturing of MNs in relevant fields. This study indicates that a printing layer thickness of 10 µm optimally balances efficiency and clinical conversion requirements. Additionally, an exposure intensity of 65 mW/cm^2^ achieves both a high fidelity and an appropriate base size. The printing angle significantly influences the morphology and mechanical properties of MNs. The diameter and aspect ratio of solid MNs correlate with their dimensional stability. Clinically, conical or quadrilateral MNs with defined parameters are recommended. A critical spacing (≥40 µm) and an optimal arrangement of the MN arrays were established. The specific exposure intensity and vertical printing angle of the hollow MNs ensure the precision of the micropore diameter and wall thickness. This approach offers theoretical insights and process parameters essential for high-precision, customizable MN engineering design.

## 1. Introduction

As a new micromedical device, microneedles (MNs) can penetrate the epidermis to the dermis without touching nerve endings or blood vessels; thus, MNs have attracted much attention in the fields of painless drug delivery and disease diagnosis and have been widely studied in many fields, such as vaccination [1], skin diseases [2], eye diseases [3], and cancer [4]. The application of polymer MNs in the biomedical field necessitates several key properties: low puncture force, high puncture depth, high puncture efficiency, painlessness, and rapid skin recovery after application. Transdermal MNs serve as a pivotal technology in modern biomedical applications, demanding meticulous optimization of their drug delivery and permeation efficiencies, as well as the extraction efficiency of interstitial fluid. For drug delivery, high drug permeation efficiency is crucial to ensure that therapeutic agents can effectively cross the skin barrier and reach target tissues while minimizing systemic side effects. Moreover, in the context of biosensing, MNs must efficiently extract interstitial fluid, which contains a wealth of biomarkers indicative of physiological states or disease conditions, with high fidelity and consistency [5,6].

The material selection of polymer MNs is crucial to their performance. MNs can be fabricated from various materials, such as silicon, metals, ceramics, and polymers. Among these, polymer MNs show significant potential in various applications because of their excellent biocompatibility, solubility, degradability, and versatility in processing methods [7,8]. The raw materials used for the preparation of polymer MNs include natural polymers, semisynthetic polymers, synthetic polymers, swellable polymers, and soluble or biodegradable polymers. For natural polymers such as starch, collagen, and silk fibroin, the prepared MNs have a low cost and nontoxic metabolites. Semisynthetic polymers such as sodium carboxymethyl cellulose have been approved for local and nonintestinal gastric medicines and have been widely used in traditional tablet and capsule drugs. Synthetic polymers, such as polyvinyl alcohol, polylactic acid, acrylic resin, etc., avoid sensitive steps in processing and are suitable for large-scale production. Common biocompatible materials include degradable polylactic acid glycolic acid copolymer, the natural polysaccharide chitosan, sodium alginate, hydrophilic hyaluronic acid, synthetic medical-grade acrylic resin, etc. When sodium alginate and hydroxyapatite MNs are printed and formed, the MN tip accuracy is relatively low because of the different equipment resolutions and printing principles [9]. Hyaluronic acid methacrylate MNs can be crosslinked by ultraviolet irradiation. However, owing to the influence of the elastic PDMS mold, the size of the MNs is smaller than that of the design model [10]. Acrylic resin has significant advantages in the preparation of high-precision MNs because of its high mechanical strength and photocuring accuracy. Although its nondegradable properties limit its long-term in vivo application, it has significant advantages in related fields, such as short-term precise drug delivery and molds that strictly control the morphology and size of MNs. The MN mold developed using acrylic resin as the main model material for MNs is compatible with most polymer MNs. Hydroxypropyl methylcellulose and polyvinylpyrrolidone (PVA) MNs, polyvinyl pyrrolidone and polyvinyl alcohol (PVA) MNs, hyaluronic acid (HA) MNs, etc., which were developed on the basis of these methods, have been applied in ocular drug delivery, Klebsiella pneumoniae biofilm control, and the treatment of ear edema [11,12,13].

These critical performance metrics of MNs are inherently intertwined with their morphological design. Xu et al. [14] demonstrated that MNs with varying tip shapes exhibit significant differences in skin penetration and that conical tips penetrate the skin more easily than pyramidal tips, exhibiting superior skin piercing capability. Anbazhagan et al. [15] used COMSOL simulations to show that conical hollow MN tips possess superior mechanical properties compared with pyramidal MN tips and that the puncture force required to penetrate the skin is linearly correlated with the diameter of the MN tip. Given a consistent base width, MNs with a height of 1000 µm are more prone to buckling than those with a height of 200 µm [16]. MNs with a diameter of 400 µm exhibit a greater breaking force and a lower puncture force than those with a diameter of 300 µm [17]. Yan et al. [18] and Gomaa et al. [19] reported that when the height of MNs is less than 600 µm, longer MNs demonstrate more effective penetration than shorter MNs. However, for MN heights exceeding 1000 µm, the penetration depth does not significantly increase with increasing height. This phenomenon is attributed to the substantial friction generated during insertion, which elevates the insertion force. Kochhar et al. [20] reported that the puncture rate for MNs with a base diameter of 200 µm (success rate: 2% to 5%) is lower than that for MNs with a base diameter of 300 µm (success rate: 1% to 52%). MN height influences the quantity of receptive cells present in the epidermis and dermis. Decreasing the height of MNs results in a lower drug dose and fewer activated cells. Increasing either the height of the MNs or the number of MNs in the MN array can enhance the drug loading capacity [21]. Optimizing the morphology of MNs can increase drug delivery efficiency. Compared with conical MNs, the groove structure at the tips of MNs facilitates drug transfer via capillary action, allowing rapid and effective deep drug delivery [22]. Self-locking MNs, characterized by a sharp tip, a wide interlocking body, and a narrow base, significantly increased adhesion to pig skin, whereas the adhesion of Christmas-tree-shaped MNs remained stable [23,24]. The likelihood of pain increases with increasing MN height. An increase in MN height from 480 µm to 1450 µm resulted in a sevenfold increase in pain sensitivity [25]. As described above, optimizing the morphological design of polymer MNs is the key to meeting their application requirements in the biomedical field.

The morphology of MNs is determined by their processing technology. The primary preparation method for polymer MNs is the micromolding, method which consists of the following steps [26,27]: (i) Preparation of the MN master mold: This method involves either micromilling to create a brass MN master mold or employing 3D printing technology to fabricate a polymer master mold [12]. (ii) Preparation of the MN negative mold: This step involves the use of polydimethylsiloxane or silicone as the material. A specific mold transfer process is employed to accurately replicate the MN master mold from step (i) onto the negative mold. (iii) MN casting and molding: The liquid polymer is injected into the MN mold via either a vacuum-assisted negative pressure process or a centrifugal method. The vacuum process utilizes the pressure differential within a vacuum environment to effectively eliminate bubbles from the liquid polymer, ensuring uniform and dense filling of the MN mold cavities. (iv) Drying and demolding: The drying process involves the removal of residual solvents or moisture from the MNs.

In the previous step (i), the most commonly used MN master molds were brass molds created through the CNC micromilling process. The brass MN mold possesses excellent mechanical properties and can be reused multiple times; however, these molds are costly to prepare and only appropriate for MN structures that have well-established morphological; their ability to produce complex MN designs is limited. Substantial differences in human skin arise from factors such as sex, region, age, and body part, along with variations in drug properties, disease characteristics, and biomarker locations. Consequently, MN morphology cannot be generalized and necessitates customized design. The preparation cost of MN molds via 3D printing is relatively low, approximately one-fifth that of brass molds for identical sizes and quantities of MNs. 3D printing enables the precise manufacture of MNs with complex customizable geometries and multifunctional capabilities, significantly broadening their application range [28,29].

The 3D printing technology used for producing MNs faces several challenges, including uncertain processing parameters, difficulties in precision control, and significant dimensional deviations during MN formation. These issues prevent the technology from meeting the stringent requirements of specific precision applications. Hollow MNs were fabricated via the liquid crystal display method, and the 3D printed height was set to approximately 1000 µm; however, the output height of curved pyramid hollow MNs was measured to be 900 ± 40 µm, and the syringe-like hollow MN height was 955 ± 40 µm [30]. A printed 3:1 aspect ratio MN with an input height of 3000 µm was 11% shorter, whereas an MN with an input height of 600 µm was more than 40% shorter than specified when a low-cost desktop SLA 3D printer was used [31]. Fitaihi et al. [12] used stereolithography 3D printing to prepare MN molds; all printed heights exhibited less than a 1% difference from the input height, whereas the tips were notably broad and blunt (85 µm size). Consequently, a discrepancy remains between the designs of current 3D printed MNs and their actual output values, presenting a significant challenge in MN manufacturing.

The standard acrylic resin investigated in this study, a fundamental component of the UV-curable material system, has exhibited remarkable application potential across diverse high-precision and consumer-oriented sectors because of its distinctive molecular architecture and photopolymerization behavior. In aerospace engineering, this material enables the fabrication of lightweight but highly accurate microsensor components, which are essential for monitoring critical parameters under extreme environmental conditions. Within the precision electronics industry, it facilitates the intricate shaping of microscale structures in chip packaging, thereby ensuring reliable signal transmission. In micromechanical manufacturing, the high-resolution molding capabilities of acrylic resin enable the production of complex components, such as microgears and hinges, with micron-level precision.

Against the backdrop of rapidly advancing additive manufacturing technologies, projection microstereolithography (PµSL), which leverages the principles of light-induced polymerization, has emerged as a pivotal technique in the fabrication of MNs and MN arrays. PµSL overcomes the limitations of traditional microfabrication methods by integrating digital projection with sequential layer-by-layer curing processes. With an optical precision of ≤10 µm, PµSL accurately replicates intricate design features, ensuring sharp MN tips and uniform array patterns. The ultrathin processing layers (≤10 µm) yield smooth surfaces, minimizing resistance during drug delivery. Moreover, the unique capability of PµSL for integrated macromicroscale fabrication streamlines the production of complex MN systems that encompass the MN body, support structures, and interconnects, thereby increasing manufacturing efficiency and structural integrity. However, current applications of PµSL in MN fabrication face significant challenges, including dimensional inaccuracies due to material shrinkage during curing, variability in morphology caused by process parameter fluctuations, and discrepancies between the designed geometries and actual fabricated structures. Thus, a comprehensive understanding of the precise control mechanisms and formation principles of PµSL-fabricated MNs is imperative for technological advancement and the translation of MN-based technologies from laboratory research to industrial-scale production.

This study focuses on standard acrylic resin, capitalizing on the high-precision capabilities of PµSL 3D printing. By employing multifaceted experimental approaches, the intricate relationships between key process variables, such as the print layer thickness, exposure intensity, and printing angle, and the resulting MN morphology and mechanical properties were systematically investigated. Through orthogonal experimental designs, this study methodically varies parameter combinations, quantifying morphological attributes such as MN height, base diameter, and tip sharpness via optical microscopy and SEM and optimizing process parameters to achieve a balance between fabrication accuracy and efficiency, enabling precise control over MN geometry. In parallel, this study explores how design parameters, including the MN profile, base diameter, aspect ratio, aperture size, and wall thickness, impact the final fabricated structures. Additionally, this study evaluates the influence of the array density and support structure design on the overall fabrication quality, providing critical insights for optimizing complex MN systems. Through rigorous experimentation and analysis, this study aims to provide foundational support for developing cost-effective, customizable acrylic resin MN prototypes, validating their structural performance and facilitating mold production.

## 2. Materials and Methods

### 2.1. Materials

A photosensitive standard acrylic resin (HTL, BMF Precision Technology Co., Ltd., Shenzhen, China), was used; its basic properties are shown in Table 1.

### 2.2. Instruments and Equipment

An additive manufacturing system (PμSL light-cured, Microarch S240, BMF Precision Technology Ltd., Shenzhen, China), a digital microscope (3DM-HK830, Aosvi, Shenzhen, China), a thermal field emission scanning electron microscope (Quanta FEG 450, FEI Company, Hillsboro, OR, USA), and a curing box (UW-03, Shenzhen Creality 3D Technology Co., Ltd., Shenzhen, China) were used.

The working principle of PμSL 3D printing involves the use of a UV light source to irradiate photosensitive resin. The resin contains molecules capable of crosslinking and curing through a photoinitiation reaction; upon exposure to the light source, these molecules rapidly crosslink and cure, resulting in the formation of a cured layer. The printing platform subsequently decreases with increasing thickness of the printing layer, and another layer of liquid resin is applied and irradiated by a light source for further curing. This process is repeated until a complete three-dimensional structure is achieved.

The preparation process is as follows: (1) Design a three-dimensional MN model via CAD software (AutoCAD 2023). (2) Convert the MN model into the STL format. (3) Send the STL file to the 3D printer and utilize slicing software (Magics, BMF customized version) to generate the printing images. (4) Fabricate the MNs layer-by-layer via a 3D printer. (5) The printed MNs were soaked in anhydrous ethanol for 10 min to remove uncured resin, rinsed thoroughly with anhydrous ethanol, and allowed to dry. (6) Finally, the MNs were placed in a curing box (UW-03, Shenzhen Creality 3D Technology Co., Ltd.) for post-treatment.

### 2.3. Printing Parameters and Shape Design Parameters of the MNs

Exposure time: Duration of exposure to UV light.

Exposure intensity: This refers to the light intensity of UV exposure, which is categorized into 256 levels ranging from 0–255.

Printing layer thickness: This refers to the thickness of each layer during the curing process. Layer thickness influences both printing speed and quality.

Printing angle: This parameter denotes the placement position or tilt angle of the MN model. An angle of 0° indicates that the MN body is positioned vertically on the printing platform, whereas 90° denotes a parallel orientation to the printing platform.

Tip size: the printer resolution is 10 µm; all MN tips are designed to be 10 µm.

Height (*H*): MN height is determined by the requirements of specific application scenarios to minimize pain, bleeding, and infection.

Base diameter (*Φ*): Considering the application height and aspect ratio, the design range for MN base diameters is 100–400 µm.

Aspect ratio (*H*:*Φ*): This parameter primarily influences the difficulty of insertion and the mechanical strength of the MNs. The recommended aspect ratio ranges from 3:1 to 5:1, with 5:1 specified for thin facial skin and 2:1 to 5:1 for deeper tissues to improve structural integrity.

MN density: Increasing the MN density permits a greater number of MNs on a single patch, thereby increasing the drug delivery capacity.

Conical and quadrangular pyramid MNs represent two fundamental geometries in MN design. Triangular pyramid and hexagonal pyramid MNs were designated as reference geometries for printing. In the design of conical MNs, four groups labeled A, B, C, and D, featuring base diameters of 100 µm, 200 µm, 300 µm, and 400 µm, and ten types of aspect ratios of 1:1, 1.5:1, 2:1, 2.5:1, 3:1, 3.5:1, 4:1, 4.5:1, 5:1, and 10:1, respectively, were created. Four different base shapes with base diameters of 300 µm were subsequently selected for printing, including conical, triangular pyramid, quadrangular pyramid cone, and hexagonal pyramid MNs with aspect ratio parameters ranging from 1:1 to 10:1. The groups were successively divided into C, E, F, and G. The conical MNs were consistent with the conical MNs in group C. All the MNs had a tip diameter of 10 µm, whereas the backing layer measured 2 mm × 2 mm × 0.6 mm.

3D printing offers distinct advantages for the fabrication of hollow MNs. The micropore diameter and wall thickness are critical design parameters that can be used to differentiate hollow MNs from solid variants. Cylindrical MNs with a diameter of 400 µm and a height of 300 µm were designed. Circular hole structures with varying diameters were incorporated into the cylindrical MNs, and the influences of the hole diameter and wall thickness on the formation quality were evaluated. The specific design parameters for the MNs are detailed in Table 2.

### 2.4. Morphology of MNs

In accordance with the appearance quality requirements for MNs outlined in the “ISO 23958-2” standard [32], the surface morphology of the MNs was examined in 3D via a high-power digital microscope, and the dimensions of the MNs, including the base diameter and height, were measured in 2D mode. SEM at a voltage of 10 kV was used to observe the thickness of the printing layer on the MN surface and the size of the tip.

### 2.5. Statistical Analysis

All the data are presented as the mean ± standard deviation (s.d.) of more than three experiments. All the statistical results presented were analyzed via one-way analysis of variance via Origin 2021 software.

## 3. Results and Discussion

### 3.1. MN Defects

**Figure 1:**
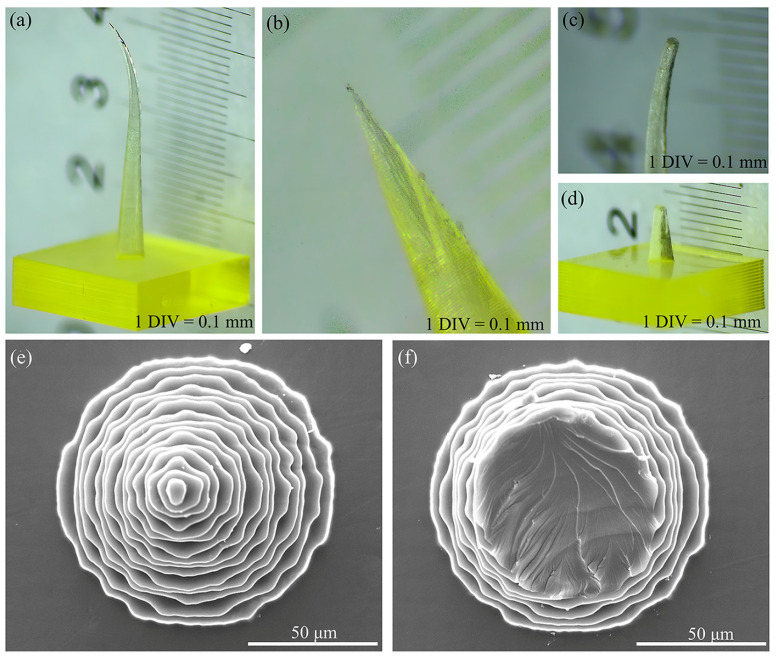
Defects of the MNs: (**a**) bent MNs; (**b**) MNs with a defect at the tip; (**c**,**d**) broken-tip MNs; (**e**) SEM image of intact MNs; and (**f**) SEM image of broken-tip MNs.

The evaluation of MNs was conducted from two perspectives: morphological integrity and dimensions. The integrity of the MN morphology was assessed, ensuring that the MN body remained upright without bending, tilting, or twisting, and confirming the absence of cracks and defects. Additionally, the sharpness, straightness, and absence of burrs or hooks at the MN tip were carefully checked. Figure 1 illustrates the common defects observed in 3D printed MNs.

Unreasonable design parameters or improper equipment operation during the 3D printing process often result in MNs that do not meet morphological expectations. For example, the bent MN body shown in Figure 1a is primarily attributed to inadequate design parameters and the processing accuracy of the equipment. When the aspect ratio of an MN is excessively large, its tip becomes slender, causing the resin to displace during the layer-by-layer curing and stacking processes, which ultimately results in bending of the MN body. In Figure 1b, the top of the MNs is absent without any visible fracture traces; this issue is primarily due to uneven adjustments of the printer platform or tension membrane during printing or insufficient resin material. The MN tips shown in Figure 1c,d exhibit clear fracture traces, and this condition typically arises when the MN tips contact foreign objects, resulting in breakage during the removal process after preparation or during postcuring operations. Figure 1e,f present SEM images of intact and broken-tip MNs, respectively.

Additionally, MN size measurement is essential. The height, base diameter, and tip size of the MNs were measured via a microscope. The actual values (output from printing) are then compared with the designed values (input for printing) to calculate the size deviation rate, thereby assessing the accuracy of the MN products.

### 3.2. Influence of Printing Layer Thickness on MN Morphology

PμSL enables MN printing through a layer-by-layer curing method, and the adjustable range of the equipment processing layer thickness is 10–40 µm. The PμSL layer thickness is affected by the accuracy of the printing platform with the tension membrane. The curing light intensity follows the Beer-Lambert law, and the theoretical layer thickness Z is smaller than the actual curing depth Dp. When Z > Dp, insufficient curing takes place, resulting in pore defects. The single-variable method was used to evaluate the influence of layer thickness on the forming quality of MNs, including printing efficiency, dimensional fidelity, and surface morphology.

D6# MNs, characterized by a height of 1400 µm, a base diameter of 30 µm, and an aspect ratio of 3.5:1, were chosen as representative MNs for printing. The numbers of layers corresponding to printing layer thicknesses of 40 µm, 30 µm, 20 µm, and 10 µm are 35 layers, 46 layers, 70 layers, and 140 layers, respectively, and the printing times for the MN body parts are 93 min, 122 min, 186 min, and 372 min, respectively. The layer thickness is significantly negatively correlated with the printing efficiency. Compared with a 10 µm layer thickness, a 40 µm layer thickness results in a 4-fold increase in efficiency.

**Figure 2:**
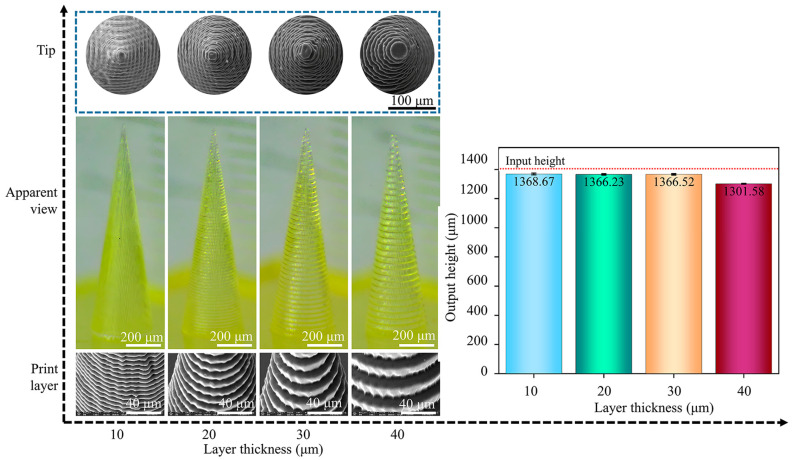
Morphology and dimensions of MNs with different printing layer thicknesses. All the data are presented as the means ± standard errors of the means (n = 3).

The morphology and dimensions of the MNs obtained when the exposure intensity was 45 mW/cm^2^ and the printing angle was 0° are shown in Figure 2. The MNs with a 10 µm layer thickness have a smooth surface and continuous interlayer transition. When the layer thickness increases to 20 µm, stepped layer lines appear in the Z axis direction, and the surface roughness exceeds the comfort threshold for human skin contact. The layer thickness has no effect on the base diameter of the MNs, which are all 400 ± 5 µm, indicating that the planar resolution of the base part is not affected by the layer thickness. The layer thickness affects both the height and the tip of the MNs. The layer thickness is positively correlated with the tip diameter. The tip diameter of the 10 µm layer-thick MNs was 10 ± 2 µm, and it increased to 35 ± 5 µm for the 40 µm layer-thick MNs. The heights of the MNs are all lower than the designed value of 1400 µm; the actual height of the 10 µm layer-thick MNs is approximately 1312 ± 25 µm, with a height fidelity of 93.71%, and the height of the 40 µm layer-thick MNs is 1245 ± 40 µm, with a height fidelity of only 92.97%. At high curing layer thicknesses, the diffusion distance of free radicals increases and the polymerization rate decreases, resulting in enhanced postcuring shrinkage.

**Figure 3:**
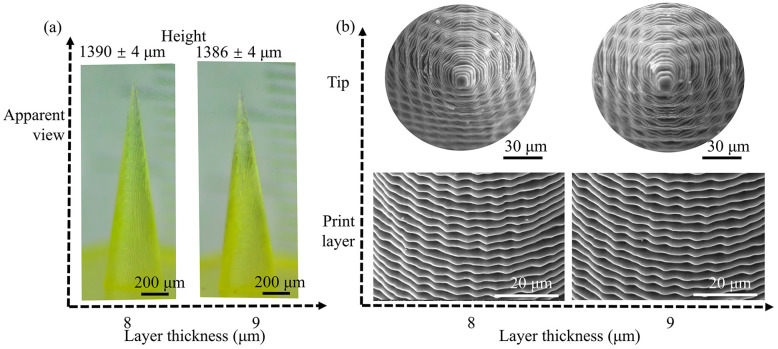
Morphology and dimensions of MNs with extreme printing layer thicknesses (8 µm, 9 µm): (**a**) output morphology and height of the MNs and (**b**) SEM image of the tip and MN body parts of the output MNs. All the data are presented as the means ± standard errors of the means (n = 3).

To explore the ultimate processing capacity of PμSL technology, the printing layer thickness was further reduced to 9 µm, 8 µm, and 7 µm, and the formation quality of the MNs was evaluated. The output morphology and dimensions are shown in Figure 3. For the 9 µm layer thickness, 156 slices were made, which took 414 min, and the MNs could be successfully prepared. The height of the MNs was approximately 1386 ± 4 µm, with a height fidelity of approximately 99.00%. For the 8 µm layer thickness, 175 slices were made. After 465 min, the corresponding MN height increased to 1390 ± 4 µm, the layer line spacing decreased to 7.95 ± 0.30 µm, and the height fidelity reached 99.28%. The surface smoothness of the 9 µm and 8 µm thick MNs was significantly improved, and the tip diameters were 9.8 ± 2 µm and 9.5 ± 1 µm, respectively.

When the layer thickness was 7 µm, the printing failed, no MN structure formed on the backing layer, and dot-like cured spots remained on the surface of the tension membrane. The uniformity of the resin layer is highly dependent on the flatness of the tensioned film and the precision of platform movement. When the layer thickness decreases to 7 µm, MN vibrations during platform displacement can induce fluctuations in the resin thickness, causing the ultraviolet irradiation dose within local regions to fall below the curing threshold, and the elastic deformation response of the polymer tensioning materials becomes more pronounced during ultrathin-layer printing, compromising the maintenance of uniform interfacial tension. This leads to spatial heterogeneity in the resin distribution, and such nonuniformity exacerbates the gradient distribution of the curing energy; the extremely reduced layer thickness magnifies subtle calibration errors in objective lens focusing, reducing the overlap rate between adjacent exposure spots below critical levels and generating discrete uncured microdomains at the resin-film interface [33,34]. The radical diffusion behavior initiated by photoinitiators under UV irradiation is highly sensitive to layer thickness. At a 7 µm thickness, the effective radical diffusion distance may become shorter than the resin layer thickness, preventing subjacent resin regions from achieving radical concentrations sufficient to initiate polymerization. Here, the combined effects of the photoinitiator concentration and UV penetration capacity fail to exceed the critical curing depth, resulting in longitudinal discontinuities in photopolymerization. Furthermore, the dramatically increased specific surface area of thin resin layers enhances the oxygen diffusion flux, causing premature termination of chain-growth reactions through radical quenching and further destabilizing the curing dynamics [35,36]. The synergistic effect of the above factors leads to 7 µm becoming the critical layer thickness of the current technology.

In terms of printing efficiency, although increasing the layer thickness significantly improved the efficiency, with a 40 µm thickness achieving fourfold higher efficiency than a 10 µm thickness, it led to a notable decline in surface quality and geometric fidelity. When the layer thickness increases to 20 µm, a stepped layer appears on the MN surface, and the surface roughness exceeds the comfort threshold for human skin contact, directly impacting the user experience. Moreover, the tip diameter increases from 10 ± 2 µm at a thickness of 10 µm to 35 ± 5 µm at a thickness of 40 µm, whereas the height fidelity decreases from 93.71% to 92.97%. For an MN tip diameter of 5 µm, the puncture force for individual MNs is approximately 0.02 N; however, this force increases to 0.167 N when the diameter reaches 37 µm [37]. The surface roughness and area of MNs significantly impact their drug loading capacity. The surface of 3D printed MNs is relatively rough due to limitations in printing accuracy, whereas microsteps create additional space for drug loading [38]. Further reducing the layer thickness enhances the surface quality and geometric fidelity: at 9 µm and 8 µm thicknesses, the MN height fidelity reaches 99.00% and 99.28%, respectively, with smaller tip diameters and smoother surfaces. However, this causes a significant drop in printing efficiency, and when the thickness is reduced to 7 µm, printing fails entirely because it falls below the critical curing depth. Overall, the 10 µm layer thickness strikes an optimal balance among key indicators: compared with thicker layers, it results in no significant step defects and maintains geometric fidelity that meets precision requirements; compared with thinner layers, it offers substantial efficiency advantages and avoids curing failure at ultralow thicknesses, addressing the dual needs for product performance and production efficiency in practical applications.

### 3.3. Influence of Exposure Intensity on MN Morphology

The exposure intensity represents the energy density of ultraviolet light received per unit area, which directly affects the decomposition rate of the photoinitiator and the crosslinking density of the resin. In this study, under the conditions of a printing layer thickness of 10 µm and an exposure time of 1 s, the influence of exposure intensity in the range of 15–85 mW/cm^2^ on the morphology and dimensions of MNs was investigated. When the exposure intensity is below 25 mW/cm^2^, the resin at the tip of the MNs is not fully cured and the edge is blurred, resulting in tip blunting. When it is greater than 35 mW/cm^2^, the light energy is concentrated, the curing boundary is clear, and the tip approaches the designed value.

Human skin consists of three different layers: the epidermis, the dermis, and the subcutaneous tissue [39]. The epidermis is divided into the stratum corneum and the active epidermis layer. The stratum corneum is the outermost layer of the skin and is composed of dense keratinocytes, with a thickness of approximately 15–20 µm. The active epidermal layer is located beneath the stratum corneum and is approximately 50–100 µm thick. It consists of a transparent layer, granular layer, spinous layer, and basal layer and contains immune active cells and a small amount of vascular-less nerve tissue. The dermis is located beneath the epidermis, is approximately 1000–3000 µm thick, and contains blood vessels, nerves, and other skin appendages. Skin thickness varies from person to person and is strongly influenced by age, sex, and the living environment [40]. The stratum corneum of scarred skin is slightly thicker than that of normal skin, and the height of MNs is greater than that of conventional MNs. Research has showed that a 1000 µm height of MNs is the optimal height for treating scarred skin MNs [41]. Owing to variations in age, sex, region, and application site, MN height designs range from 100–2000 µm, with facial designs spanning 100–1000 µm, whereas deeper treatments may reach 2000 µm.

**Figure 4:**
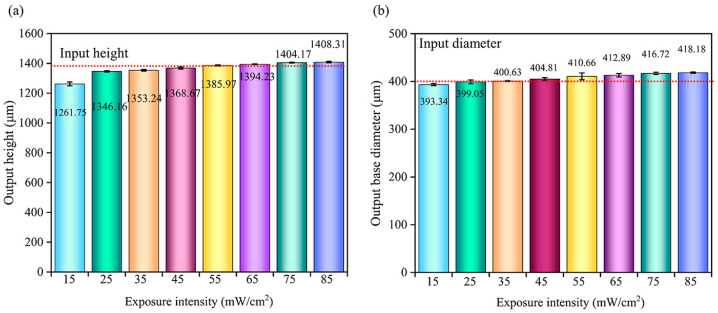
Influence of exposure intensity on the output dimensions of MNs: (**a**) influence on the output height; and (**b**) influence on the output base diameter. All the data are presented as the means ± standard errors of the means (n = 3).

The height of the MNs is dominated by the depth of light penetration and the interlayer bonding force. As the exposure intensity increases from 15 mW/cm^2^ to 85 mW/cm^2^, the average height of the MNs significantly increases from 1261.75 µm to 1408.31 µm, indicating a positive correlation, as shown in Figure 4a. When the exposure intensity is less than 25 mW/cm^2^, the light energy is insufficient to drive complete crosslinking of the resin, resulting in an insufficient single-layer curing depth, and the height of the MNs is significantly lower than the designed value. When it is above 35 mW/cm^2^, the resin is fully crosslinked, the curing depth matches the designed layer thickness, the interlayer bonding is tight, and the height gradually approaches the designed value. When it reaches 75 mW/cm^2^, the height exceeds the designed value, indicating that the light energy has fully penetrated into the deep layer of the resin. When the exposure intensity is extremely high, overcuring of the resin occurs, further leading to the height deviation from the designed value.

A larger base size of MNs helps reduce the displacement generated when it pierces the skin and lowers the possibility of MN fracture. Furthermore, since the force at the base mainly acts on the epidermis, it does not increase the pain. Studies have shown that there are no significant differences in pain scores among MN volunteers with heights of 700 µm and base widths of 160 µm, 245 µm, and 465 µm [25]. The substrate size affects the drug loading because a large amount of surface area and volume are dispersed at the bottom of the MNs. The change in the base diameter is shown in Figure 4b. As the exposure intensity increased, the base diameter gradually increased from 393.34 µm (10 mW/cm^2^) to 418.18 µm (85 mW/cm^2^). When the exposure intensity is 35 mW/cm^2^, the base diameter approaches the designed value. The lateral diffusion of ultraviolet light in the resin causes the cured area to exceed the designed pattern, and the base diameter increases with increasing exposure intensity. The size of the base diameter is determined by the balance between light scattering and resin shrinkage.

According to the MN design requirements, an exposure intensity range of 35–75 mW/cm^2^ is preferred. In this range, the tip shape, height, and base diameter of the MNs can be close to the design value. For example, if the height of the MNs is slightly higher, the exposure intensity can be appropriately increased, but not more than 75 mW/cm^2^; if the base dimensions of the MNs are precisely matched to the design value, the exposure intensity can be set at approximately 35 mW/cm^2^. Another strategy for dealing with a particular shape of MNs is to perform a reverse design; that is, the design height is greater than the target height, and the design base diameter is smaller than the target value to balance the final MN shape.

During the process of preparing MNs via 3D printing, the thermal effect of acrylic resin and the curing kinetics form a coupling effect through the mechanism of “light absorption, cross linking heat release, and stress response”, which directly determines the morphological accuracy and dimensional stability of the MNs [42,43]. The energy density, which is the core regulatory parameter, governs the decomposition rate of the photoinitiator and the crosslinking depth of the resin via the Beer-Lambert law: When the exposure intensity is less than 25 mW/cm^2^, the input of light energy is insufficient. The concentration of free radicals generated by the decomposition of the photoinitiator cannot trigger complete crosslinking, resulting in incomplete curing of the resin at the tip of the MN. The edges that have not been fully crosslinked present a blurred state, forming a tip passivation phenomenon. At this time, the exothermic effect of the resin is weak, and the thermal shrinkage effect can be ignored. When between 35 and 75 mW/cm^2^, light energy is converted into chemical energy to drive the efficient crosslinking of the double bonds of acrylate monomers. The crosslinking density increases with increasing exposure intensity. The accompanying exothermic effect increases the temperature of the resin, accelerates the diffusion of free radicals, and ensures that the curing depth precisely matches the designed layer thickness (10 µm). The height of the MNs linearly approaches the design value as the crosslinking depth increases. The transverse curing expansion caused by light scattering and the thermal contraction of the resin form a dynamic equilibrium, making the substrate diameter approach the design value (400 µm) at 35 mW/cm^2^. When the exposure intensity is greater than 75 mW/cm^2^, the penetration depth of the light energy exceeds the layer thickness. Excessive curing causes an internal temperature gradient in the resin, and the shrinkage stress generated by the saturation of the surface crosslinking density exceeds the tensile strength of the material. The height of the MNs exceeds the design value because of additional deep-layer curing. At the same time, high temperature intensifies the lateral scattering of ultraviolet light, causing the substrate diameter to increase significantly as the light scattering expansion effect surpasses contraction compensation (from 400 µm to 418.18 µm).

On the basis of the above coupling mechanism, the critical thresholds for exposure intensity are defined as follows: 35 mW/cm^2^ serves as the effective curing initiation value to ensure that the free radical concentration exceeds the critical threshold required for the polymerization reaction; 75 mW/cm^2^ is set as the runaway critical value to avoid dimensional deviations caused by excessive curing. In view of the different influences of exposure intensity on the MN height and substrate diameter, the corresponding design strategies are as follows: When prioritizing the MN height, 50–60 mW/cm^2^ is selected to balance the curing depth and thermal stress; when precise control of the substrate diameter is needed, 35–40 mW/cm^2^ is set to utilize the counteracting effect of light scattering expansion and thermal contraction. For complex morphological requirements, the reverse design—increase the design height by 5% and reduce the substrate diameter by 3% relative to target values—compensates for the final dimensions through excessive curing and scattering expansion during high-intensity exposure to ensure coordinated optimization of MN tip sharpness, height accuracy, and substrate size.

### 3.4. Influence of the Printing Angle on MN Morphology

**Figure 5:**
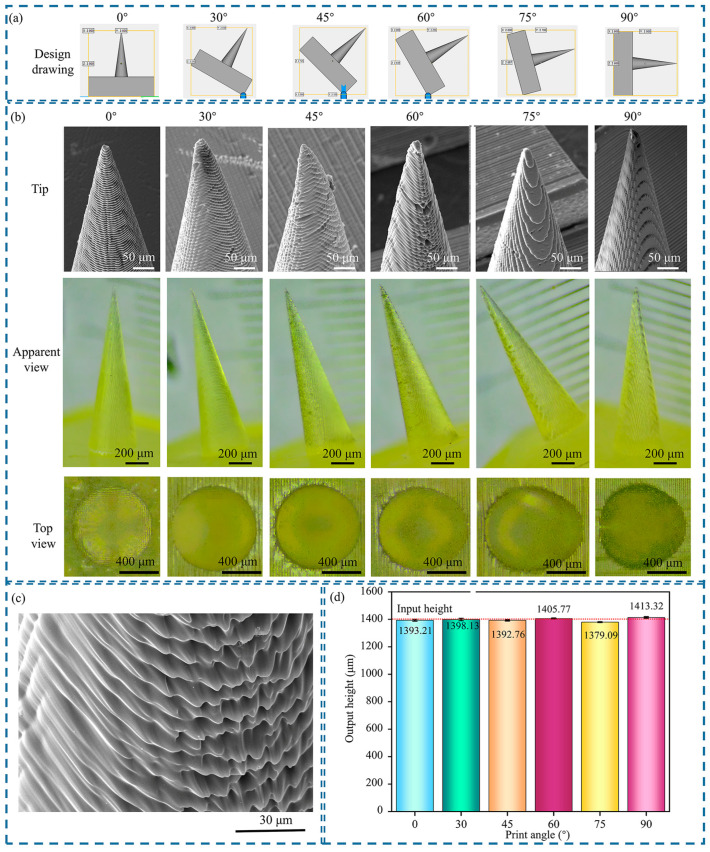
Morphology and height change diagram of conical MNs with different printing angles: (**a**) schematic diagram of the printing angle; (**b**) output morphology diagram of the MNs; (**c**) local enlarged view of the MNs after 60° printing; and (**d**) influence of the printing angle on the height of the MNs. All the data are presented as the means ± standard errors of the means (n = 3).

The printing angles of the MNs (0°, 30°, 45°, 60°, 75°, and 90°) were designed in the slicing software, and support structures were added, as shown in Figure 5a. The support structures are only used to maintain the stability of the MN components during the printing process and are removed after printing. The number of slices corresponding to different printing angles for the MN part is 140, 131, 113, 97, 55, and 40 in sequence. An increase in the printing angle reduces the number of layers of the printed MN part. However, for the square backing layer, after inclined printing, the total number of slices is 200, 287, 262, 267, 236, and 200 in sequence. The number of slices is the smallest for 0° and 90°, and the printing time is the shortest.

With a printing layer thickness of 10 µm and an exposure intensity of 65 mW/cm^2^, the prepared MN morphology is shown in Figure 5b,c. The MNs prepared at all printing angles are perpendicular to the backing layer. The MN bodies at 0° and 90° are straight without bending, and the tips are intact. For the other printing angles, the MN tips are slightly bent. When the MNs are printed at low angles (0° and 30°), the central axis of the MNs is approximately perpendicular to the printing platform, the layer-by-layer stacking direction is consistent with the tip growth direction, the projection area of each layer is uniform, the stress distribution between the cured layers is symmetrical, and the MN surface is smooth without defects. When printing at medium-high angles (45°, 60°, 75°), the inclination causes a change in the cross-sectional area of a single layer, and the optical path of ultraviolet light in the resin is extended, triggering an interlayer step effect. In addition, the uncured resin sags and flows on one side of the MN body under the action of gravity, resulting in the drooping deformation of the cured layer edge and a rough MN surface. When the printing angle increased to 90°, the central axis of the MNs was parallel to the printing platform, the cured layer was relatively uniform, and the MN morphology remained intact.

As the printing angle increased, the base shape of the MNs gradually changed from circular to asymmetrical ovals. The inclined printing causes the resin to be affected by the interlayer shear force and surface tension, resulting in deviation of the cured edge. The aspect ratio of the base increases from 1.0 to 1.3. The influence of the printing angle on the height of the MNs is shown in Figure 5d. When the printing angle is 75°, the height of the MNs is the smallest, and when the printing angle is 90°, the height of the MNs is the largest, with a height difference of approximately 34 µm. In addition, it was observed in the experiment that the MNs printed at a 45° angle did not have a significant advantage in height, which is inconsistent with the situation reported in the literature [44], that the height of conical MNs printed at 45° is significantly greater than that of 0° and 90° outputs. This difference is related to the resin properties, support structure design, and post treatment process.

The surface roughness and base shape changes were caused by the printing angle. The various MNs replicated via the 3D printing master mold continue to replicate their surface structure, resulting in changes in the interaction between the newly prepared MNs and the skin. This influence is particularly significant in the relationship between the surface roughness of MNs and the drug loading and puncture force. When the printing angle changes, the surface roughness of the MNs changes accordingly, and this change affects the drug loading and puncture force through multiple mechanisms. From the perspective of drug loading capacity, an increase in surface roughness can significantly enhance the drug loading capacity of MNs. When the printing angle is in the medium to high range, a distinct stepped texture appears on the surface of the MNs. This rough surface structure significantly increases the contact area between the MNs and drugs, allowing drug molecules to be more fully adsorbed and embedded in these microscopic grooves and protrusions, thereby significantly enhancing the drug loading capacity of the MNs. In addition, rough surfaces can further stabilize the loading state of drugs and reduce drug leakage during storage and transportation by altering the physicochemical interactions between drugs and MNs, such as increasing van der Waals forces and hydrogen bonds. In terms of the puncture force, the surface roughness is positively correlated with the puncture force. The smooth surface of MNs results in less friction with the skin tissue during the puncture process, which enables them to penetrate the skin more smoothly and requires a relatively lower puncture force. However, as the printing angle changed, resulting in an increase in surface roughness, the frictional resistance between the MNs and the skin tissue during puncture significantly increased. When the rough surface of the MNs comes into contact with the skin, the microscopic protrusions have stronger mechanical interactions with the skin tissue, hindering the smooth insertion of the MNs and thereby significantly increasing the puncture force. Printing at medium and high angles (45–75°) can also cause the base shape of MNs to become elliptical. This shape change has a negative effect on the mechanical properties of MNs in the skin, thereby increasing the stability of the drug channel. When MNs are inserted into the skin, the uniformity of force application is crucial for maintaining their structural integrity and function. The elliptical base breaks the original symmetrical structure of the MNs, causing the forces borne by different parts of the MNs to no longer be evenly distributed when they are squeezed and rubbed by the skin tissue. During the insertion process, the force in the long axis direction is greater, whereas the force in the short axis direction is smaller. This uneven force distribution will cause stress concentration within the MNs. The structural stability of MNs, especially those with relatively high heights, is relatively weak. This stress concentration is more likely to cause bending deformation of the MNs. Once the MNs bend, the originally designed drug channels are distorted or even blocked, severely affecting the drug release path and release efficiency. The drug may not be released into the target tissue at the expected speed and dosage, resulting in a significant reduction in the therapeutic effect. In addition, curved MNs may also generate abnormal stress within the skin, further damaging the skin tissue and triggering adverse reactions.

### 3.5. Design and Output Morphology of Conical Solid MNs

During the MN design process, the base diameter (*Φ*) and height (*H*) were taken as key factors, and a series of conical solid MNs were prepared accordingly. The base diameters covered four specifications: 100 µm, 200 µm, 300 µm, and 400 µm, and the aspect ratios (*H*:*Φ*) were set as 1:1, 1.5:1, 2:1, 2.5:1, 3:1, 3.5:1, 4:1, 4.5:1, 5:1, and 10:1.

In Group A (base *Φ*100 µm), A1 MNs with a height of only 100 µm could be successfully fabricated. However, the three MNs (A1, A2, and A3) with aspect ratios below 2:1 had tips that were not prominent. The A9 MNs, with an aspect ratio of 5:1, maintained an upright position, but more than 50% of the A9 MNs experienced tip loss. A10 MNs, with an aspect ratio of 10:1, are relatively rare in practical applications and were designed to investigate the processing limits of the equipment used in this study. In the A10 group, 80% of the MNs presented bent tips and tip loss defects. This high defect rate can be attributed to the small base diameter, which resulted in inadequate interlayer bonding strength of the acrylic resin during the curing process.

In Group B (base *Φ*200 µm), the tips of the B1 MNs, which had an aspect ratio of 1:1, were clear and distinct. The B9 MNs, with an aspect ratio of 5:1, were designed to have a height of 1000 µm, adequately fulfilling the standard height requirement for MNs. Among the B10 MNs, which have an aspect ratio of 10:1, more than 50% exhibited bending beginning at the midpoint, resulting in offset tips. An increase in the aspect ratio was correlated with a higher probability of output defects. Although the MNs with an aspect ratio of 10:1 attained the expected height, an uneven internal stress distribution resulted from curing shrinkage, causing a notable inclination of the tip.

In Group C (base *Φ*300 µm), all MNs with an aspect ratio below 5:1 were successfully printed. The C10 MNs, characterized by an aspect ratio of 10:1 and a designed height of 3000 µm, are classified as ultrahigh MNs, which is uncommon in practical research. The large base diameter mitigated the instability associated with the high aspect ratio, resulting in the C10 MNs maintaining an upright MN body and a clear tip.

In Group D (base *Φ*400 µm), all MNs with an aspect ratio below 10:1 were perfectly printed. Notably, MNs with an aspect ratio above 4:1 are classified as ultrahigh MNs, which are used solely to assess the printing capabilities of the equipment.

An increase in the aspect ratio results in a reduction in the tip curvature radius of the MNs. The shrinkage stress from resin curing accumulates at the tip and leads to fractures. In MNs with a small base diameter and large aspect ratio, the stress field during resin curing is uneven across a small cross-section. When the shear force at the MN tip exceeds the material’s yield strength, total deformation occurs.

**Figure 6:**
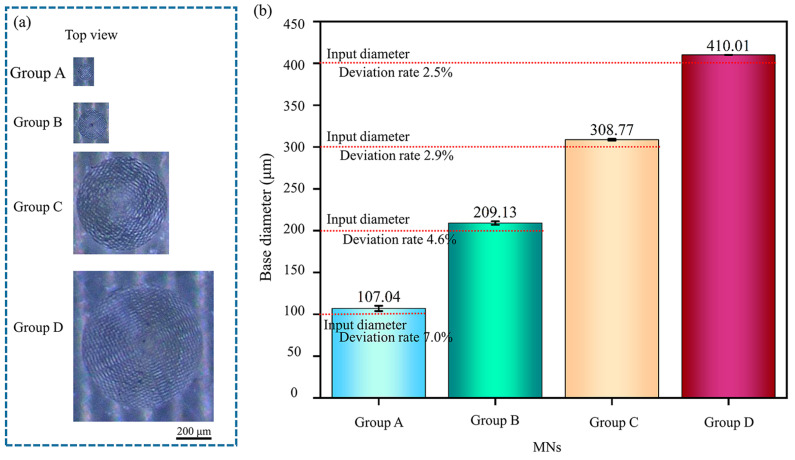
Comparison of base diameter sizes and input/output values of conical solid MNs: (**a**) top view of MNs and (**b**) comparison of input and output values of MN bases. All the data are presented as the means ± standard errors of the means (n = 3).

Figure 6 shows the input and output values of the MNs with varying base diameters. A smaller base diameter of the MNs corresponds to a higher deviation rate. The deviation rate for a base diameter of *Φ*100 µm is approximately 7.0%; however, as the base diameter increases to 400 µm, this rate decreases to 2.5%. The trend in the base diameter deviation rate confirms the interaction between materials, energy, and structure in PμSL 3D printing. For small-sized bases, the curing time of the resin is inadequate, leading to an unbalanced state postcuring. During the printing of the subsequent layer, it is challenging for the liquid resin at the edge of the cured layer to completely detach from the cured area, which results in increased size deviation. As the base diameter increases, the stress-bearing area of the resin expands, resulting in a more uniform material distribution and effective dispersion of ultraviolet light, thereby reducing the base diameter deviation rate.

**Figure 7:**
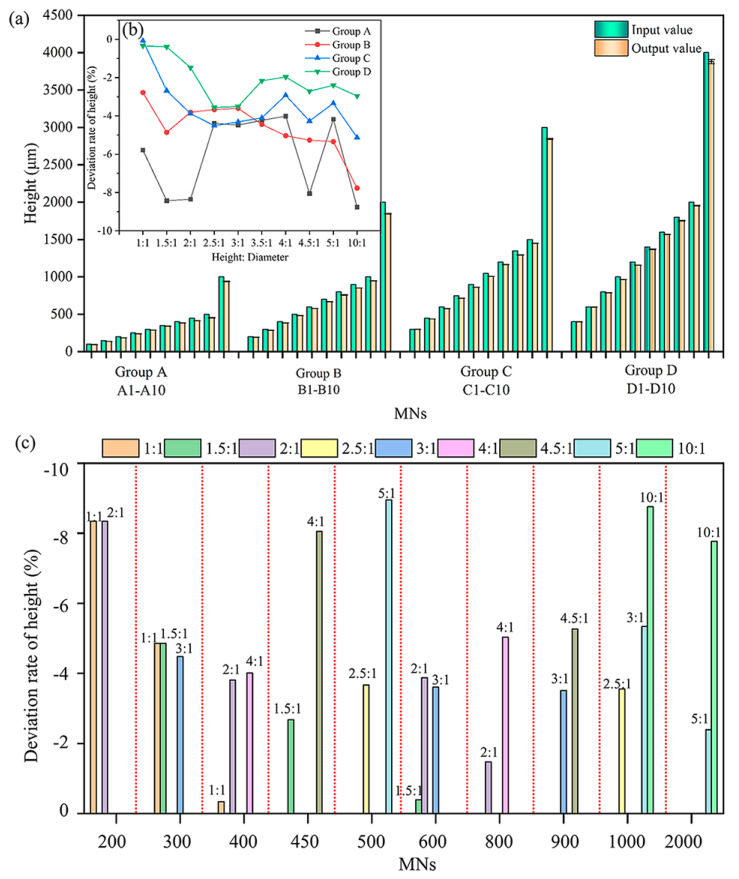
Comparison of the height and input/output values of conical solid MNs: (**a**) comparison of the input and output height values of the MNs; (**b**) output height deviation rate diagram of the MNs; and (**c**) influence of the aspect ratio on the output height of conical solid MNs. All the data are presented as the means ± standard errors of the means (n = 3).

Figure 7a shows that, under the current processing parameters, all the MNs exhibit output heights lower than the designed values because of curing shrinkage. In PμSL technology, ultraviolet light cross links the monomer, leading to the rearrangement of molecular chains and resulting in volume shrinkage. This anisotropic shrinkage produces a cumulative effect along the height of the conical MNs. Figure 7b presents the output height deviation rates at varying aspect ratios. Generally, an increase in base diameter (e.g., *Φ*400 µm) results in a larger inter-layer contact area of the photocured resin, reducing the interlayer stress concentration and promoting more uniform curing shrinkage. Consequently, the absolute values of the height deviation rates are lower (all less than 4%). The *Φ*100 µm MNs exhibit uneven curing shrinkage due to their small base area and light scattering effects, resulting in a height deviation rate of up to −9%. The base diameter influences structural stability by adjusting the moment of inertia of the cross-section. The optimal aspect ratio ranges are 2.5:1–4:1 for *Φ*100 µm, 1:1–4.5:1 for *Φ*200 µm, and 1:1–10:1 for *Φ*300–400 µm.

The output height deviation rates of the MNs with different aspect ratios under the same height design are shown in Figure 7c. According to material mechanics theory, the critical buckling load of an MN is inversely proportional to the square of its aspect ratio. A larger base diameter effectively enhances the critical buckling load by increasing the support area. At a constant height, variations in the aspect ratio correspond to differences in the base diameter. The base diameter influences the deviation rate by affecting the inter-layer contact area and stress distribution. Consequently, as the aspect ratio increases, the cumulative effect of curing shrinkage along the MN height increases, leading to an increased height deviation rate.

Through orthogonal experimental analysis of four combinations of substrate diameters (*Φ*100, 200, 300, and 400 µm) and ten combinations of aspect ratios (1:1–10:1), combined with the process characteristics of PμSL technology, the morphological design parameter range of acrylic resin solid MNs was established. From the perspective of experimental failure modes, the influence of the substrate diameter on the formation quality of MNs is highly variable: When the aspect ratio of the substrate diameter of *Φ*100 µm MNs exceeds 4:1 (such as A9 MNs with an aspect ratio of 5:1), more than 50% of the MNs have tip loss, whereas for A10 MNs with an aspect ratio of 10:1, due to insufficient interlayer bonding force, 80% have tip bending or loss. The upper limit of the aspect ratio of the *Φ*200 µm substrate was increased to 4.5:1, but the B10 MNs with an aspect ratio of 10:1 still exhibited middle bending and tip offset of 50% due to uneven curing shrinkage stress. The *Φ*300~400 µm substrate, owing to its moment of inertia being 81~256 times greater than that of *Φ*100 µm (based on Euler’s formula, the critical buckling load is positively correlated with the moment of inertia), can support stable printing with an aspect ratio of 10:1 (for example, the defect rate of C10 and D10 MNs is <5%). This shows the effective offsetting ability of large substrates against the instability risk caused by high aspect ratios.

Based on the defect thresholds, process feasibility, and functional adaptability observed in the comprehensive experiments, the parameter range established in Table 3 not only avoids the insufficient interlayer bonding force and curing defects caused by the excessive aspect ratio of small sizes but also meets the verification requirements of the equipment’s extreme processing capacity under large sizes. This provides a reliable parameter window for the subsequent engineering applications of MN arrays in fields such as precise drug delivery and biosensing.

### 3.6. Design and Output Morphology of the Pyramidal Solid MNs

Conical, triangular pyramid, quadrangular pyramid, and hexagonal pyramid MNs were chosen because of their representation of the fundamental morphologies of MNs with single vertices in triangular, quadrilateral, and hexagonal shapes. Section 3.5 presents a comprehensive evaluation of *Φ*300 µm conical solid MNs. In this section, triangular pyramid, quadrangular pyramid, and hexagonal pyramid MNs with an inscribed circle of *Φ*300 µm were printed and analyzed. MNs with an inscribed circle of *Φ*300 µm and aspect ratios ranging from 1:1 to 10:1 were successfully produced. As the angular regions of the pyramids cure, material shrinkage in three orthogonal directions occurs, leading to an increase in local stress. The triangular pyramid MNs (angle of 120°) and quadrangular pyramid MNs (angle of 90°) have a larger overlapping area in the photocuring paths of adjacent prism faces. The resin shrinkage stress induces a self-locking effect at the edges, minimizing pixelation defects and resulting in sharp edges. The angle between adjacent prism faces of the hexagonal pyramid MNs is 60°, and the spot diameter of PμSL is 10 µm, closely matching its edge curvature radius of approximately 5 µm. This projection results in discrete pixel points at the edges, where the overlapping rate of adjacent exposure areas is inadequate, leading to blurred edges.

**Figure 8:**
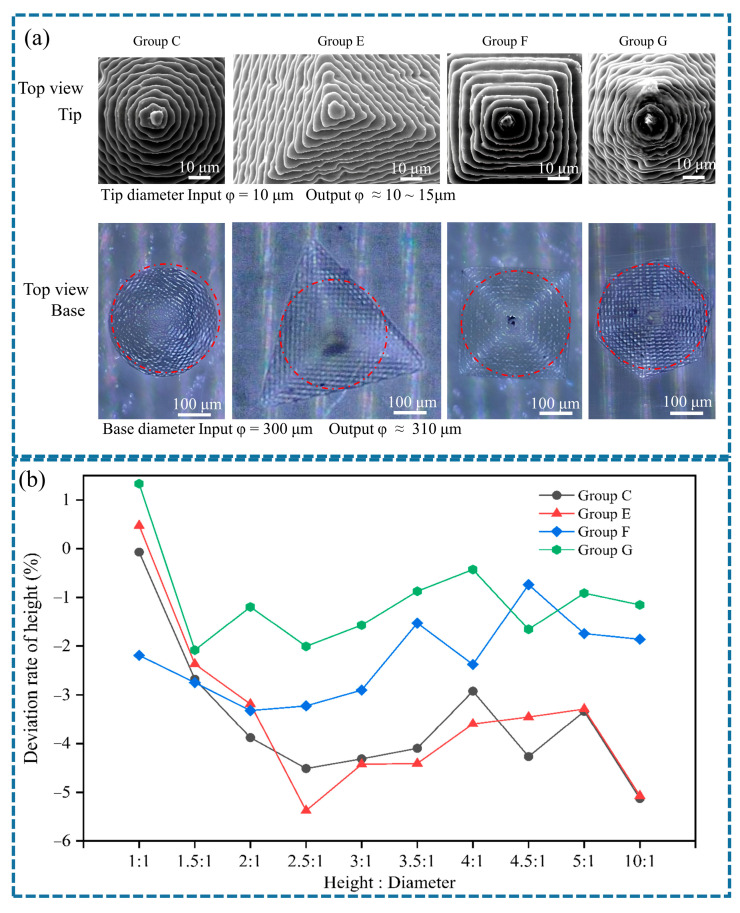
Comparison of the output sizes of MNs in group C, group E, group F, and group G: (**a**) top view of the tip and base parts; (**b**) output height deviation diagram.

The mask projection of PμSL presents a pixelated pattern. The step effect at the edge of the inscribed circle results in an actual cured diameter that exceeds the designed value, as illustrated in Figure 8a,b. The tip sizes of both conical and pyramidal MNs are comparable, measuring approximately 10–15 µm. The measured value of the inscribed circular base is 310 ± 10 µm, with an absolute deviation rate of 3.3% from the designed value of 300 µm.

The output heights of the four MN types were analyzed, and the results are displayed in Figure 8b. At an aspect ratio of 1:1, the output height of the pyramidal MNs is slightly greater than the expected value. For aspect ratios between 2.5:1 and 5:1, the height deviation rate remains relatively stable. Within this range, the height deviation rates, listed from smallest to largest, are as follows: hexagonal pyramidal, quadrangular pyramidal, conical, and triangular pyramidal. The triangular pyramidal MNs feature fewer sides and lower structural symmetry, causing stress to concentrate at the edges. Consequently, it has the highest output height deviation rate, reaching −5.37%. The continuous curvature of the conical MNs directs the resin’s shrinkage during the curing process along the axial direction, resulting in significant axial shrinkage accumulation. Compared with conical MNs with a single curve, quadrangular pyramidal and hexagonal pyramidal MNs have high symmetry, allowing for an even distribution of shrinkage stress and a less pronounced accumulation effect. Therefore, their height deviation rates are lower.

### 3.7. MN Array Design and High-Density Output Strategy

In MN arrays, a higher MN density means that there are more MNs within a unit area, which directly increases the contact area with the skin and provides more penetration channels for the drug. For example, in vaccination applications, high-density MN arrays can enable antigens to enter the epidermis and dermis more rapidly and widely, thereby effectively stimulating an immune response. Moreover, an appropriate MN spacing is equally crucial for drug delivery. Overly dense MN spacing may cause MNs to interfere with each other when inserted into the skin, resulting in some MNs being unable to penetrate the skin completely and instead reducing the efficiency of drug delivery. However, if the MN spacing is too large, it will reduce the number of contact sites between the drug and the skin and fail to fully leverage the advantages of the MN array. The density of MNs and the spacing significantly influence the degree of skin damage and the healing process. Although high-density MN arrays can increase drug delivery capacity, they can also cause more tiny wounds to the skin, which may trigger a more intense inflammatory response and slow the speed of skin healing. If the MN spacing is too small, the skin will be subjected to excessive MN punctures in a short period of time, which may also cause excessive damage to the skin tissue and increase the risk of infection. In contrast, appropriately reducing the density of MNs and increasing the MN spacing can reduce the area of skin damage and promote faster skin healing. In practical applications, it is necessary to precisely regulate the MN density and MN spacing on the basis of the therapeutic objective and the skin’s recovery ability to ensure the drug delivery effect while reducing adverse effects on the skin. For MN arrays used in biosensing, MN density and MN spacing play important roles in their accuracy. A higher MN density can increase the contact area with biological samples and increase the sample collection volume, thereby providing more abundant information for biosensing and contributing to improved detection accuracy and sensitivity. However, if the MN spacing is unreasonable, it may lead to overlapping or cross-contamination of the collected samples, affecting the reliability of the test results. An appropriate MN spacing ensures that each MN independently collects samples, avoiding interference between samples and guaranteeing the accuracy and stability of the biosensing data.

To improve the transdermal application efficiency of MNs, multiple MNs are typically arranged in an array format within a patch. Research has indicated that high-density MN arrays can expand the contact area with the skin, thereby effectively increasing the drug loading capacity. This section investigates the minimum critical spacing between adjacent MNs in conical MN arrays with varying base diameters and compares the maximum MN densities in square and hexagonal arrangements.

**Figure 9:**
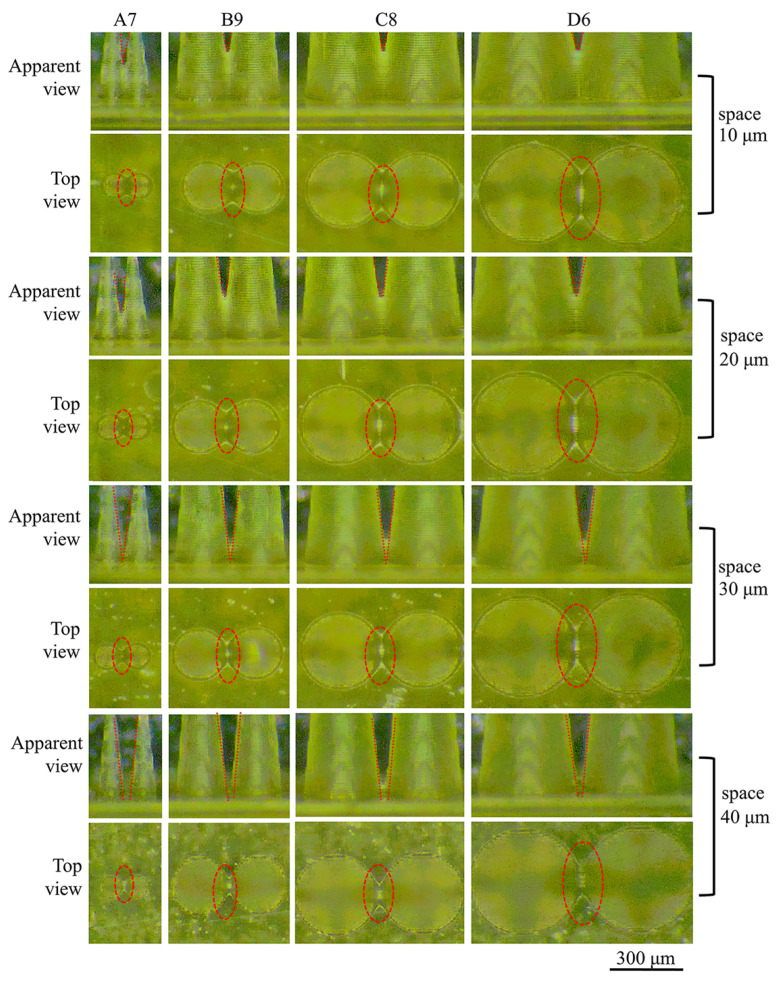
Output morphology of conical MN arrays with different spacing (the red dashed lines in the figure indicate the overlapping areas of adjacent MNs).

According to geometric morphology theory, when the base diameter is fixed, an increase in the aspect ratio results in a smaller tip curvature radius of the MNs. This change increases the horizontal projection area of adjacent MNs and increases the likelihood of overlap due to geometric constraints. As the base diameter decreases, the contact area between the MNs diminishes. However, the influence of surface tension becomes more pronounced, leading to a further reduction in the angle between adjacent MNs. By integrating the results from Table 4 with practical applications, the MNs with the largest aspect ratios from groups A–D (specifically A7, B8, C8, and D6) were chosen as research models. The formation characteristics of MN arrays with adjacent spacing of 10 µm, 20 µm, 30 µm, and 40 µm were examined, and the results are shown in Figure 9. At spacing of 10 µm and 20 µm, adjacent MNs exhibit significant overlap due to geometric interference from the tip curvature, with overlap heights of approximately 180 µm and 130 µm, respectively. As the spacing between adjacent MNs increases to 30 µm, the stress distribution becomes more uniform, leading to a significant decrease in the overlap height to approximately 20 µm. When the spacing is increased to 40 µm, microscopic imaging reveals that adjacent MNs are distinctly separated and do not interfere with one another. We recommend maintaining an MN spacing of at least 40 µm to prevent overlap.

The arrangement of conical MNs on the patch dictates the MN density. This study focuses solely on the ability to process high-density MNs. We calculated the number of MNs in both the square and hexagonal array configurations. As an example, the results of the use of a 1 cm^2^ square MN patch are presented in Table 4. The hexagonal array consistently accommodates 15.7% to 17.4% more MNs than the square array through longitudinal optimization. Compared with the square array, the spacing has a greater effect on the MN density in the hexagonal array. If a high-density arrangement is necessary, the hexagonal array offers greater space utilization. The formulas for the density calculation are provided in Formulas (1) and (2).

(1) $$N_{\mathrm{conical}\text{-}\mathrm{Squre}}\approx {\left(\frac{\mathrm{Square}\;\mathrm{side}\;\mathrm{length}}{\mathrm{Base}\;\mathrm{diameter}+\mathrm{MNs}\;\mathrm{spacing}}\right)}^{2}$$

(2) $$N_{conical\text{-}\mathrm{Hex}}\approx \left(\frac{\mathrm{Square}\;\mathrm{side}\;\mathrm{length}-\mathrm{Base}\;\mathrm{diameter}}{\mathrm{Base}\;\mathrm{diameter}+\mathrm{MNs}\;\mathrm{spacing}}\right)\times \left[\frac{\mathrm{Square}\;\mathrm{side}\;\mathrm{length}}{\left(\mathrm{Base}\;\mathrm{diameter}+\mathrm{MNs}\;\mathrm{spacing}\right)\times \frac{\sqrt{3}}{2}}\right]$$

**Figure 10:**
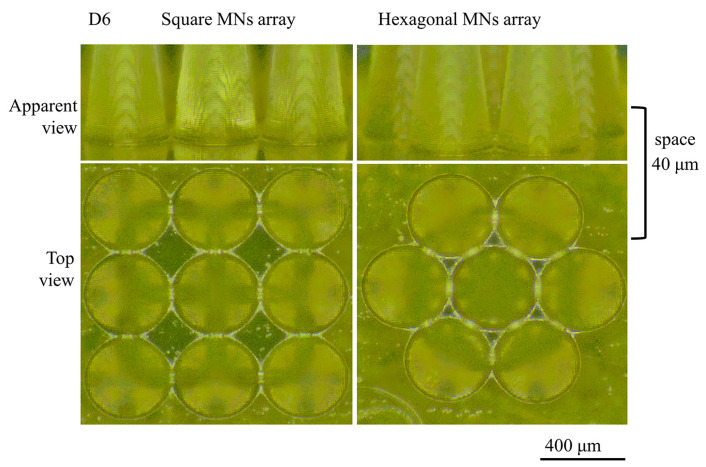
Output morphologies of square and hexagonal arrays of D6 MNs.

Using the D6 sample as a model, we utilized square and hexagonal arrays for printing with adjacent MN spacings of 40 µm; the resulting morphology is displayed in Figure 10.

In designing pyramidal MN arrays, the initial step is to achieve high-density arrangements of MNs by optimizing the space-filling efficiency of the base pattern. Additionally, the geometric constraints of adjacent MN base patterns, such as the minimum distances between vertices and tips, vertices and edges, and edges, must be strictly controlled. The critical factor in designing various MN arrays is the choice of the close packing method for the base pattern. For triangular pyramid MNs, employing a honeycomb close packing arrangement of equilateral triangle bases maximizes the number of MNs per unit area. Pyramidal MNs attain structural stability through an orthogonal grid arrangement of squares. The base of the hexagonal pyramid MN features a hexagonally close packed structure that ensures a high packing density and exhibits isotropic mechanical properties.

Using a 1 cm^2^ square MN patch as an example, we observed that with an adjacent MN spacing of 40 µm, the maximum density distributions of the MNs in groups E, F, and G are presented in Table 5.

The variation laws of the number of pyramidal MNs accommodated in a square MN patch are shown in Formulas (3)–(5):

(3) $$N_{\mathrm{Triangular}\;\mathrm{pyramid}}\approx \left(\frac{2\times \mathrm{Square}\;\mathrm{side}\;\mathrm{length}}{\left(\mathrm{Base}\;\mathrm{diameter}+\mathrm{MNs}\;\mathrm{spacing}\right)\times \sqrt{3}}\right)\times \left[\frac{2\times \mathrm{Square}\;\mathrm{side}\;\mathrm{length}}{\left(\mathrm{Base}\;\mathrm{diameter}+\mathrm{MNs}\;\mathrm{spacing}\right)\times 3}\right]$$

(4) $${N\;}_{\mathrm{Quadrangular}\;\mathrm{pyramid}}\approx {\left(\frac{\mathrm{Square}\;\mathrm{side}\;\mathrm{length}}{\mathrm{Base}\;\mathrm{diameter}+\mathrm{MNs}\;\mathrm{spacing}}\right)}^{2}$$

(5) $$N_{\mathrm{Hexagonal}\;\mathrm{pyramid}}\approx \left(\frac{\mathrm{Square}\;\mathrm{side}\;\mathrm{length}-\mathrm{Base}\;\mathrm{diameter}}{\mathrm{Base}\;\mathrm{diameter}+\mathrm{MNs}\;\mathrm{spacing}}\right)\times \left[\frac{\mathrm{Square}\;\mathrm{side}\;\mathrm{length}}{\left(\mathrm{Base}\;\mathrm{diameter}+\mathrm{MNs}\;\mathrm{spacing}\right)\times \frac{\sqrt{3}}{2}}\right]$$

**Figure 11:**
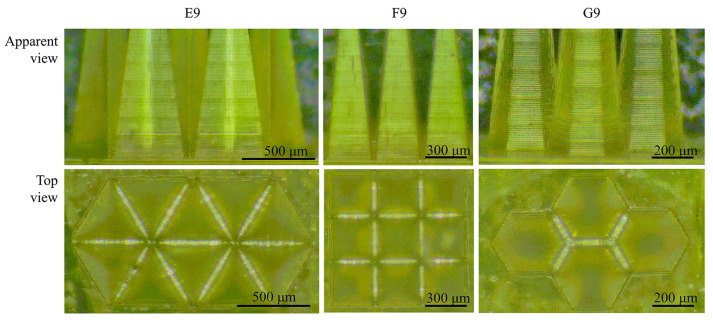
Output morphologies of typical close packed MN arrays in groups E, F, and G.

The #9 MNs in groups E, F, and G were designed and arranged via the aforementioned closest packing method, ensuring a minimum spacing of 40 µm between adjacent MNs for the printing process. Figure 11 shows the morphological characteristics of the closely packed arrays of 9# MNs (aspect ratio 5:1) for each group. The pyramid, spaced 40 µm apart, can be printed independently, featuring distinct edges and corners for each MN and well-defined boundaries at their bases.

### 3.8. Influence of the Processing and Design Parameters of Hollow MNs on the Output Morphology

At high exposure intensities, both the curing depth and critical energy of the photosensitive resin increase. This expansion allows for greater lateral diffusion of the resin, resulting in a cured area that surpasses the intended design specifications. Excessive exposure may lead to overcuring of the material surrounding the circular holes, resulting in shrinkage or deformation. This, in turn, adversely affects the dimensions and through-hole properties of the circular holes. For example, the HD10 MNs, characterized by a pore diameter of 100 µm, were printed with a layer thickness of 10 µm, a printing angle of 0°, and an exposure intensity varying between 15 and 85 mW/cm^2^. When the exposure intensity is below 35 mW/cm^2^, insufficient curing occurs, leaving some materials to polymerize. Consequently, while micropores can form, the overall height of the MNs remains relatively low. At an exposure intensity of 45 mW/cm^2^, the pore diameter is approximately 99.08 µm. The level of curing achieved at this intensity is suitable, allowing the hollow MNs to maintain the intended pore size effectively. A further increase in the exposure intensity enhances the light scattering effect, leading to overcuring of the resin in the through-holes region, which consequently results in a gradual decrease in the pore diameter. At an exposure intensity of 75 mW/cm^2^, the micropores become completely closed. Considering the HT50 sample, which has a wall thickness of 50 µm and employs the same processing parameters as the HD10 sample, the average wall thickness gradually increases from 50.51 µm to approximately 55.26 µm as the exposure intensity increases. Therefore, when hollow MNs are printed, a lower exposure intensity enhances the accuracy of both the pore diameter and wall thickness.

**Figure 12:**
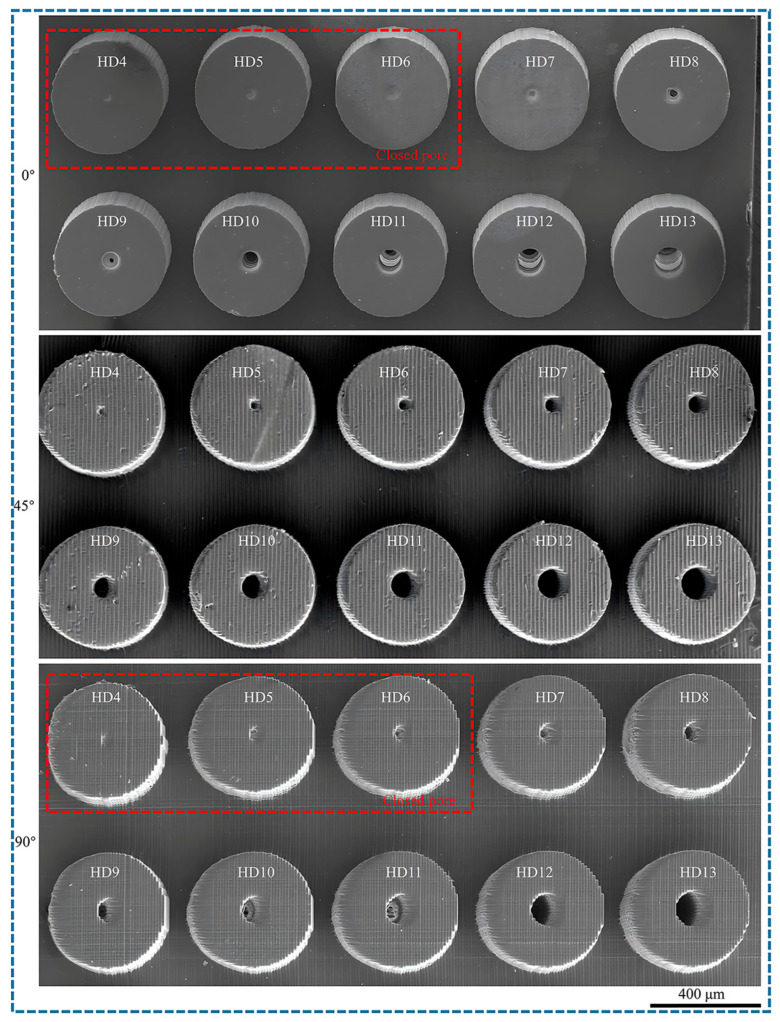
SEM images of the output morphologies of the micropores in Group H at different printing angles.

Hollow microcolumns were printed with pore diameters between 40 and 130 µm and wall thicknesses from 5–50 µm, utilizing a layer thickness of 10 µm, an exposure intensity of 45 mW/cm^2^, and printing angles of 0°, 45°, and 90°. The morphologies of the microcylinders with varying pore diameters are displayed in Figure 12. HD4 corresponds to a hole with a diameter of 40 µm, whereas D13 corresponds to a hole with a diameter of 130 µm. At a printing angle of 0°, micropores smaller than 60 µm are not fully formed because of the effects of curing diffusion and weak interlayer bonding. Although micropores with diameters between 70 and 90 µm are formed, their sizes are relatively small. However, micropores larger than 100 µm can achieve full vertical penetration. At a printing angle of 45°, micropores larger than 50 µm can progressively achieve penetration. The uneven distribution of the interlayer stress results in minor angular deviations in the micropores. At a printing angle of 90°, the interlayer is perpendicular to the MN axis. Micropores smaller than 60 µm are not formed, those between 70 and 110 µm achieve gradual penetration, and larger micropores above 120 µm can achieve complete formation because of the extensive support area and all micropores exhibit significant angular deviations.

**Figure 13:**
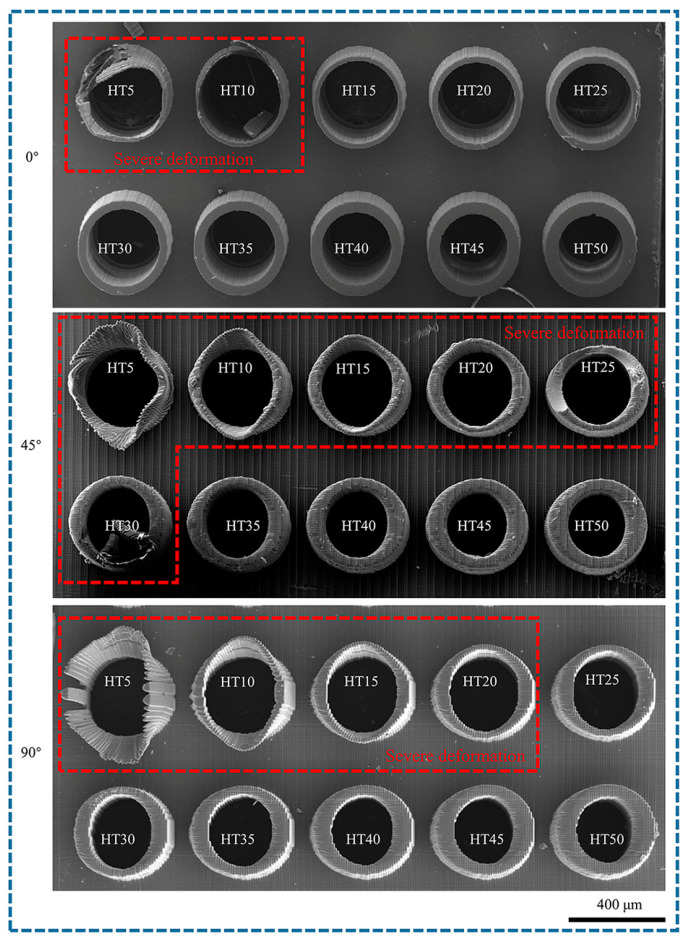
SEM images of the thin-wall output morphology of group H at different printing angles.

**Figure 14:**
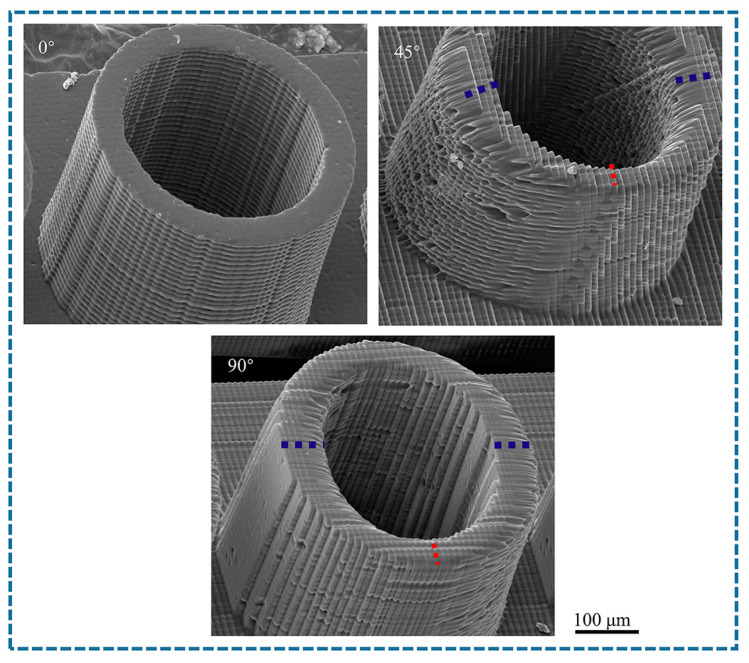
SEM images of the outer surfaces of the hollow MNs in group H at different printing angles (the purple dotted line represents the wall thickness of the area parallel to the printing layer, and the red dotted line represents the wall thickness of the area perpendicular to the printing layer).

The morphologies of the different wall thicknesses are displayed in Figure 13. The 5 µm thick walls exhibited significant deformation and cracking. Wall thicknesses greater than 15 µm at a printing angle of 0° can be successfully printed; however, thin walls exceeding 20 µm can align perpendicularly with the patch distribution while maintaining stable output. At a printing angle of 45°, thin walls with thicknesses of 5–30 µm experience significant deformation, whereas those thicker than 35 µm become misaligned. At a printing angle of 90°, deformation of the thin wall is most pronounced. The 5 µm thin wall experiences complete cracking, whereas the 10–25 µm thin walls exhibit significant displacement. Thin walls exceeding 25 µm can be successfully printed; however, their shapes are misaligned. The printing angle significantly affects the wall thickness. Figure 14 shows the wall thickness variations at different angles.

When the printing angle is 0°, the layer-by-layer stacking direction aligns with the axis of the MNs, resulting in minimal suspended structures and uniform wall thickness without any offset. In contrast, thin walls printed at other angles exhibit uneven wall thicknesses due to suspended printing. The section perpendicular to the printing layer has a smaller wall thickness, whereas the section parallel to the printing layer has a larger wall thickness. Consequently, the section perpendicular to the printing layer is more susceptible to deformation. The micropores and thin walls printed at a 45° angle demonstrate strong forming capability; however, they exhibit large holes and numerous defects on the outer surface of the cylinder. This resembles the appearance of conical MNs printed at the same angle, as noted in Section 3.4, thereby presenting a challenge for designing hollow MN channels.

The internal chamber size of hollow MNs is influenced by both material properties and manufacturing processes, typically ranging from 30–300 µm, and their customizable design facilitates the efficient delivery of large molecule drugs [45,46]. For example, pyramid-shaped hollow MNs with a diameter of 100 µm have successfully delivered vitamin B12 in vitro and insulin in vivo [47]. Additionally, a pyramid-shaped MN array with a pore size of 200 µm and a conical hollow MN with a diameter of 100 µm can effectively extract skin interstitial fluid and monitor glucose, pH, and lactic acid in situ [48,49]. According to the Hagen-Poiseuille equation, fluid resistance is directly proportional to channel length and inversely proportional to the fourth power of the radius. Increased angle deviation prolongs the actual flow path, which can decrease the flow rate or alter the flow pattern. If deviations cause asymmetry in the channel’s cross-section, they may result in drug retention or vortices, which can alter drug distribution in subcutaneous tissue. The dermal anisotropy of the skin further amplifies the effect of angular deviation. In precision medical scenarios, angular deviations may lead to differences in therapeutic effects or the risk of side effects. It is necessary to optimize the insertion parameters in combination with the individual characteristics of patients. Considering the printing accuracy of the equipment, we summarized the design ranges for the pore diameter and wall thickness corresponding to various printing angles for a hollow MN design, as presented in Table 6.

## 4. Conclusions

This study systematically investigated the design, manufacturing, and performance evaluation of acrylic resin MNs via PμSL technology. By regulating the morphological parameters and array configurations, we identified the causes of MN size deviations and proposed control strategies. This research offers a theoretical foundation and a set of process parameters for the engineering design of high-precision, customizable MNs. Additionally, it serves as a valuable reference for developing MN prototypes and manufacturing-associated molds in areas such as transdermal drug delivery and biosensing. The conclusions are as follows:

(1) The printing layer thickness is pivotal for balancing efficiency and accuracy. When the printing speed is increased from 10 µm to 40 µm, the surface roughness and skin comfort are increased. Thickness is negatively correlated with MN height and tip diameter. A 10 µm thickness, chosen as the benchmark, ensures 94% height fidelity, reduces the insertion force, and facilitates precise drug delivery, optimizing both manufacturing and biological performance.
(2) Exposure intensity is critical, as it governs resin crosslinking and curing, shaping MN morphology. Below 25 mW/cm^2^, tip passivation occurs, whereas over 75 mW/cm^2^ leads to overcuring. An optimal 65 mW/cm^2^ ensures 99.57% fidelity, yielding sharp tips and ideal heights for painless skin penetration and efficient drug delivery. Deviations disrupt the MN structure, impairing insertion and drug release.
(3) The printing angle critically shapes MN morphology and mechanics. Vertical printing (0°) yields smooth, high-fidelity MNs, ensuring stable drug delivery. Mid–high angles (45–75°) induce a stepping effect and deformation, creating oval bases and height reduction, risking breakage and inconsistent penetration. A 90° angle increases the height but demands extra support. These morphological changes also impact biological performance, affecting drug delivery and biosensing efficiency.
(4) The base diameter and aspect ratio are pivotal for the dimensional stability of solid MNs. A larger base diameter minimizes shrinkage, enhancing mechanical support during skin penetration. When the aspect ratio exceeds 5:1, structural failure becomes more likely. Conical MNs contract more axially, whereas pyramid-shaped MNs experience more uniform stress.
(5) Geometric analysis and experiments revealed that a minimum 40 µm spacing and close hexagonal arrangement optimize MN array performance. This setup boosts the density by 15–17% compared with square arrays while preventing geometric interference. Pyramidal honeycombs maximize density, and orthogonal pyramidal meshes enhance stability. For transdermal delivery, hexagonal conical or pyramidal arrays balance drug loading and skin tolerance, optimizing biological performance.
(6) The fidelity of the pore diameter and wall thickness of hollow MNs hinges on the exposure intensity and printing angle. A 45 mW/cm^2^ exposure and 0° printing angle minimize deviation, ensuring precise pore formation and structural integrity. Nonvertical angles cause offsets and uneven walls. Controlled pore size and uniform thin walls are vital for efficient drug release, fluid extraction, and mechanical stability in biological applications.

In summary, compared with traditional 3D printing technologies, PµSL enables high-precision MN fabrication through the adjustment of processing and design parameters. This capability lays the groundwork for the development of precise and complex MN structures. This study did not explore MN arrays beyond their basic morphology in depth. Future work should focus on establishing a mapping model between print parameters and MN morphology to facilitate the large-scale production of complex MN devices via PµSL technology. Moreover, owing to significant disparities in light source uniformity, mechanical precision, and printing materials among PµSL systems from various manufacturers, coupled with constraints imposed by manufacturing costs, cross-device repeatability verification was not conducted in this study.

## Figures and Tables

**Table 1:** Basic properties of standard acrylic resin (HTL).

Viscosity (25 °C) (mPa·s)	Tensile Strength (MPa)	Elongation at Break (%)	Elastic Modulus (GPa)	Bending Strength (MPa)	Bending Modulus (GPa)	Hardness (Shore D)	Coefficient of Thermal Expansion (50–100 °C) (µm/m/°C)
85	72	8	2.4	113	2.8	81	169

**Table 2:** Specific design parameters for the MNs.

Group	Shape	Base Diameter (µm)	Solid MN Number: Height (µm) (Corresponding Aspect Ratio) Hollow MN Number: Micropore Diameter (µm) (Wall Thickness) (µm)
A	Solid Conical	100	A1: 100 (1:1), A2: 150 (1.5:1), A3: 200 (2:1), A4: 250 (2.5:1), A5: 300 (3:1), A6: 350 (3.5:1), A7: 400 (4:1), A8: 450 (4.5:1), A9: 500 (5:1), A10: 1000 (10:1)
B		200	B1: 200 (1:1), B2: 300 (1.5:1), B3: 400 (2:1), B4: 500 (2.5:1), B5: 600 (3:1), B6: 700 (3.5:1), B7: 800 (4:1), B8: 900 (4.5:1), B9: 1000 (5:1), B10: 2000 (10:1)
C		300	C1: 300 (1:1), C2: 450 (1.5:1), C3: 600 (2:1), C4: 750 (2.5:1), C5: 900 (3:1), C6: 1050 (3.5:1), C7: 1200 (4:1), C8: 1350 (4.5:1), C9: 1500 (5:1), C10: 3000 (10:1)
D		400	D1: 400 (1:1), D2: 600 (1.5:1), D3: 800 (2:1), D4: 1000 (2.5:1), D5: 1200 (3:1), D6: 1400 (3.5:1), D7: 1600 (4:1), D8: 1800 (4.5:1), D9: 2000 (5:1), D10: 4000 (10:1)
E	Solid Triangular Pyramid	Inscribed circle diameter 300	E1: 300 (1:1), E2: 450 (1.5:1), E3: 600 (2:1), E4: 750 (2.5:1), E5: 900 (3:1), E6: 1050 (3.5:1), E7: 1200 (4:1), E8: 1350 (4.5:1), E9: 1500 (5:1), E10: 3000 (10:1) Group F (Quadrangular pyramid) and Group G (Hexagonal pyramid) were numbered according to the same rules as group E.
H	Hollow Cylinder	300	Cylinder height 300 µm, hollow, aperture distributions were as follows: HD4: 40, HD5: 50, HD6: 60, HD7: 70, HD8: 80, HD9: 90, HD10: 100, HD11: 110, HD12: 120, HD13: 130; HT5: 390 (5), HT10: 380 (10), HT15: 370 (15), HT20: 360 (20), HT25: 350 (25), HT30: 340 (30), HT35: 330 (35), HT40: 320 (40), HT45: 310 (45), HT50: 300 (50)

**Table 3:** Morphological parameter design range of acrylic resin solid MNs prepared by PµSL.

Aspect Ratio (*H:Φ*)	*Φ*100 µm	*Φ*200 µm	*Φ*300 µm	*Φ*400 µm
1:1	×	○	√	√
1.5:1	×	√	√	√
2:1	×	√	√	√
2.5:1	√	√	√	√
3:1	√	√	√	√
3.5:1	√	√	√	√
4:1	√	√	√	○
4.5:1	√	√	√	○
5:1	×	√	√	○
10:1	×	×	○	○

Symbols in the table: × indicates poor processing feasibility; ○ indicates that it can be successfully printed, but the MN height exceeds the transdermal medical range, and it is recommended to use it with caution; √ indicates both processing feasibility and practicality, and it is recommended for use.

**Table 4:** Maximum density of conical MN arrays with different base diameters.

Base Diameter	Number of MNs Distributed on 1 cm^2^ Square Patch		Advantages of Hexagonal Arrays over Quadrilateral Arrays
	Quadrilateral Array	Hexagonal Array	
100 µm	83 × 83 = 6889	83 × 96 = 7968	15.7%
200 µm	45 × 45 = 2025	45 × 52 = 2340	15.6%
300 µm	31 × 31 = 961	31 × 36 = 1116	16.1%
400 µm	23 × 23 = 529	23 × 27 = 621	17.4%

**Table 5:** Maximum distribution densities of the pyramidal MN arrays.

MNs Array	Base Inscribed Circle Diameter	Number of MNs Distributed on 1 cm^2^ Square Patch
Triangular pyramid	100 µm	96 × 55 = 5280
	200 µm	52 × 30 = 1560
	300 µm	36 × 20 = 720
	400 µm	27 × 15 = 405
Quadrangular pyramid	100 µm	83 × 83 = 6889
	200 µm	45 × 45 = 2025
	300 µm	31 × 31 = 961
	400 µm	23 × 23 = 529
Hexagonal pyramid	100 µm	83 × 96 = 7968
	200 µm	45 × 52 = 2340
	300 µm	31 × 36 = 1116
	400 µm	23 × 27 = 621

**Table 6:** Different printing angles corresponding to the design ranges of the aperture and wall thicknesses.

Print Angle		0°	45°	90°
Micropore diameter	Impenetrability	<60 µm	<40 µm	<60 µm
	Offset and through	-	≥50 µm	≥70 µm
	Vertical distribution and complete penetration	≥100 µm	-	-
Wall thickness	Severe deformation	≤10 µm	≤30 µm	≤20 µm
	Slight deformation	15–20 µm	35–40 µm	25–35 µm
	Complete (deformation does not affect application)	≥25 µm	≥45 µm	≥40 µm

## Data Availability

The original contributions presented in this study are included in the article, and further inquiries can be directed to the corresponding authors.

## References

[1] Sheng T., Luo B.W., Zhang W.T., Ge X.Y., Yu J.C., Zhang Y.Q., Gu Z. (2021). Microneedle-mediated vaccination: Innovation and translation. Adv. Drug Deliv. Rev..

[2] Han Y.H., Qin X.Y., Lin W.S., Wang C., Yin X.Y., Wu J., Chen Y., Chen X., Chen T. (2025). Microneedle-based approaches for skin disease treatment. Nano-Micro Lett..

[3] Wang L., Guo Y.S., Chen B., Lu S., Yang J., Jin Y., Wang X., Sun X., Wang S., Wang B. (2025). An annular corneal microneedle patch for minimally invasive ophthalmic drug delivery. Sci. Adv..

[4] Xue H.Y., Jin J., Huang X., Tan Z., Zeng Y.S., Lu G., Hu X., Chen K., Su Y., Hu X. (2025). Wearable flexible ultrasound microneedle patch for cancer immunotherapy. Nat. Commun..

[5] Lin Y.X., Dervisevic M., Yoh H.Z., Guo K., Voelcker N.H. (2025). Tailoring design of microneedles for drug delivery and biosensing. Mol. Pharm..

[6] Visscher M., Frijlink H.W., Hinrichs W.L.J. (2025). What is the optimal geometry of dissolving microneedle arrays? A literature review. Pharmaceutics.

[7] Jia B.L., Xia T.D., Wang X.H., Xu Y., Guo Z. (2023). Morphology design of polymer microneedle arrays: Key factors from the application perspective. J. Drug Deliv. Sci. Technol..

[8] Rajput A., Kulkarni M., Deshmukh P., Pingale P., Garkal A., Gandhi S., Butani S. (2021). A key role by polymers in microneedle technology: A new era. Drug Dev. Ind. Pharm..

[9] Wu M.X., Zhang Y.J., Huang H., Li J., Liu H., Guo Z., Xue L., Liu S., Lei Y. (2020). Assisted 3D printing of microneedle patches for minimally invasive glucose control in diabetes. Mater. Sci. Eng. C.

[10] Chang H., Zheng M.J., Yu X., Than A., Seeni R.Z., Kang R., Tian J., Khanh D.P., Liu L., Chen P. (2017). A swellable microneedle patch to rapidly extract skin interstitial fluid for timely metabolic analysis. Adv. Mater..

[11] Vinayakumar K.B., Silva M.D., Martins A., Mundy S., González-Losada P., Sillankorva S. (2023). Levofloxacin-loaded microneedles produced using 3D-printed molds for *Klebsiella Pneumoniae* biofilm control. Adv. Ther..

[12] Fitaihi R., Abukhamees S., Chung S.H., Craig D.Q.M. (2024). Optimization of stereolithography 3D printing of microneedle micro-molds for ocular drug delivery. Int. J. Pharm..

[13] Tunçel E., Tort S., Han S., Yücel Ç., Tırnaksız F. (2025). Development and optimization of hydrogel-forming microneedles fabricated with 3d-printed molds for enhanced dermal diclofenac sodium delivery: A comprehensive in vitro, ex vivo, and in vivo study. Drug Deliv. Transl. Res..

[14] Xu N., Zhang M., Xu W., Ling G., Yu J., Zhang P. (2022). Swellable PVA/PVP hydrogel microneedle patches for the extraction of interstitial skin fluid toward minimally invasive monitoring of blood glucose level. Analyst.

[15] Anbazhagan G., Suseela S.B., Sankararajan R. (2023). Effect of hollow microneedle geometry structure on mechanical stability and microfluidic flow for transdermal drug delivery applications. Microfluid. Nanofluid..

[16] Moussi K., Bukhamsin A., Hidalgo T., Kosel J. (2019). Biocompatible 3d printed microneedles for transdermal, intradermal, and percutaneous applications. Adv. Eng. Mater..

[17] Razzaghi M., Seyfoori A., Pagan E., Askari E., Hassani Najafabadi A., Akbari M. (2023). 3D printed hydrogel microneedle arrays for interstitial fluid biomarker extraction and colorimetric detection. Polymers.

[18] Yan G., Warner K.S., Zhang J., Sharma S., Gale B.K. (2010). Evaluation needle length and density of microneedle arrays in the pretreatment of skin for transdermal drug delivery. Int. J. Pharm..

[19] Gomaa Y.A., Morrow D.I., Garland M.J., Donnelly R.F., El-Khordagui L.K., Meidan V.M. (2010). Effects of microneedle length, density, insertion time and multiple applications on human skin barrier function: Assessments by transepidermal water loss. Toxicol. Vitr..

[20] Kochhar J.S., Quek T.C., Wei J.S., Choi J., Shui Z., Kang L. (2013). Effect of microneedle geometry and supporting substrate on microneedle array penetration into skin. J. Pharm. Sci..

[21] Römgens A.M., Bader D.L., Bouwstra J.A., Oomens C.W. (2016). Predicting the optimal geometry of microneedles and their array for dermal vaccination using a computational model. Comput. Methods Biomech. Biomed. Eng..

[22] Xu R.G., Guo H.L., Chen X.L., Xu J., Gong Y., Cao P., Wei C., Xiao F., Wu D., Chen W. (2023). Smart hydrothermally responsive microneedle for topical tumor treatment. J. Control. Release.

[23] Joo S.H., Kim J., Hong J., Lahiji S.F. (2022). Dissolvable self-locking microneedle patches integrated with immunomodulators for cancer immunotherapy. Adv. Mater..

[24] Huang D.Q., Fu X., Zhang X.X., Yuanjin Z. (2023). Christmas tree-shaped microneedles as folfirinox spatiotemporal delivery system for pancreatic cancer treatment. Research.

[25] Gill H.S., Denson D.D., Burris B.A., Prausnitz M.R. (2008). Effect of microneedle design on pain in human volunteers. Clin. J. Pain.

[26] Saifullah K.M., Mushtaq A., Azarikhah P., Prewett P.D., Davies G.J., Faraji Rad Z. (2025). Micro-vibration assisted dual-layer spiral microneedles to rapidly extract dermal interstitial fluid for minimally invasive detection of glucose. Microsyst. Nanoeng..

[27] Mei X., Zhu D.S., Li J., Huang K., Hu S., Xing M., Cheng K. (2025). Minimally invasive snakebite inspired microneedle delivery system for internal organs. Bioact. Mater..

[28] Prajapati B.G., Alzaghari L.F., Alam P., Fareed M., Kapoor D.U. (2025). Revolutionizing neurological therapies: The role of 3D printed microneedles in precision brain targeted drug delivery. J. Drug Deliv. Sci. Technol..

[29] Jia B.L., Xia T.D., Xu Y.T., Li B. (2025). Staggered design of UV-curable polymer microneedle arrays with increased vertical action space. Polymers.

[30] Xenikakis I., Tsongas K., Tzimtzimis E., Katsamenis O., Demiri E., Zacharis C., Georgiou D., Kalogianni E., Tzetzis D., Fatouros D. (2022). Transdermal delivery of insulin across human skin in vitro with 3D printed hollow microneedles. J. Drug Deliv. Sci. Technol..

[31] Krieger K.J., Bertollo N., Dangol M., Sheridan J.T., Lowery M.M., O’Cearbhaill E.D. (2019). Simple and customizable method for fabrication of high-aspect ratio microneedle molds using low-cost 3D printing. Microsyst. Nanoeng..

[32] (2022). Traditional Chinese medicine—Dermal needles for Single Use—Part 2: Roller-Type.

[33] Ge Q., Li Z.Q., Wang Z.L., Kowsari K., Zhang W., He X., Zhou J., Fang N.X. (2020). Projection micro stereolithography based 3D printing and its applications. Int. J. Extrem. Manuf..

[34] Huang J.J., Zhang B.W., Xiao J.F., Zhang Q.L. (2022). An approach to improve the resolution of DLP 3D printing by parallel mechanism. Appl. Sci..

[35] Guo T., Xia M., Yang W., Na Q., Zhang J., Yao W., Yang F., Luo Y. (2022). Model of UV-curing thickness for new thiol-ene resin for additive manufacturing of energetic materials. Addit. Manuf..

[36] Levato R., Dudaryeva O., Garciamendez-Mijares C.E., Kirkpatrick B.E., Rizzo R., Schimelman J., Anseth K.S., Chen S., Zenobi-Wong M., Zhang Y.S. (2023). Light-based vat-polymerization bioprinting. Nat. Rev. Methods Primers.

[37] Wei H.J., Liu S., Tong Z.X., Chen T., Yang M., Guo Y., Sun H., Wu Y., Chu Y., Fan L. (2022). Hydrogel-based microneedles of chitosan derivatives for drug delivery. React. Funct. Polym..

[38] Yang Q.L., Zhong W.Z., Liu Y.W., Hou R., Wu Y., Yan Q., Yang G. (2023). 3D-printed morphology-customized microneedles: Understanding the correlation between their morphologies and the received qualities. Int. J. Pharm..

[39] Mostafavi Yazdi S.J., Baqersad J. (2022). Mechanical modeling and characterization of human skin: A review. J. Biomech..

[40] Liu X., Cleary J., German G.K. (2016). The global mechanical properties and multi-scale failure mechanics of heterogeneous human stratum corneum. Acta Biomater..

[41] Zhang Q., Shi L., He H., Liu X., Huang Y., Xu D., Yao M., Zhang N., Guo Y., Lu Y. (2022). Down-Regulating Scar Formation by Microneedles Directly via a Mechanical Communication Pathway. ACS Nano.

[42] Jiang F., Drummer D. (2020). Curing kinetic analysis of acrylate photopolymer for additive manufacturing by photo-DSC. Polymers.

[43] Cai R., Luo X., Xie G., Wang K., Peng Y., Rao Y. (2024). Effects of the printing parameters on geometric accuracy and mechanical properties of digital light processing printed polymer. J. Mater. Sci..

[44] Economidou S.N., Pissinato Pere C.P., Okereke M., Douroumis D. (2021). Optimisation of design and manufacturing parameters of 3D printed solid microneedles for improved strength, sharpness, and drug delivery. Micromachines.

[45] Dardano P., De Martino S., Battisti M., Miranda B., Rea I., De Stefano L. (2021). One-shot fabrication of polymeric hollow microneedles by standard photolithography. Polymers.

[46] Kim J., Jeong J., Jo J.K., So H. (2025). Hollow microneedles as a flexible dosing control solution for transdermal drug delivery. Mater. Today Bio.

[47] Ghate V., Renjith A., Badnikar K., Pahal S., Jayadevi S.N., Nayak M.M., Vemula P.K., Subramanyam D.N. (2023). Single step fabrication of hollow microneedles and an experimental package for controlled drug delivery. Int. J. Pharm..

[48] Abbasiasl T., Mirlou F., Mirzajani H., Bathaei M.J., Istif E., Shomalizadeh N., Cebecioğlu R.E., Özkahraman E.E., Yener U.C., Beker L. (2024). A wearable touch-activated device integrated with hollow microneedles for continuous sampling and sensing of dermal interstitial fluid. Adv. Mater..

[49] Cheng J.L., Huang J.K., Xiang Q., Dong H. (2023). Hollow microneedle microfluidic paper-based chip for biomolecules rapid sampling and detection in interstitial fluid. Anal. Chim. Acta.

